# Microbialite Diversity and Ocean Redox Geochemistry of the Late Tonian Callison Lake Formation

**DOI:** 10.1111/gbi.70054

**Published:** 2026-06-08

**Authors:** Charlotte Spruzen, Malcolm W. Wallace, Maxwell A. Lechte, Ashleigh V. S. Hood, Brennan O'Connell, Galen P. Halverson

**Affiliations:** ^1^ Department of Earth and Planetary Sciences/GEOTOP McGill University Montréal Quebec Canada; ^2^ School of Earth Sciences University of Melbourne Parkville Victoria Australia; ^3^ Department of Earth Sciences University of Cambridge Cambridge UK

**Keywords:** carbonate, dolomite, neoproterozoic, ocean redox, oxygenation, thrombolite

## Abstract

Microbialites provide a unique insight into ancient microbial processes and environments, but trends in the diversity of unlaminated microbialites remain poorly understood. The ca. 745 Ma Callison Lake Formation in the Yukon (northwest Canada) features a range of microbialites which are diverse at both the mesoscale and microscale. Four microbialite facies are recognised from the Ramp member of the Callison Lake Formation, all of which include framework structures with syn‐depositional cavities. The thrombolite facies of the Callison Lake Formation is typified by exceptional preservation, with isopachous primary marine cements and distinct microclots that we suggest formed through early, mimetic carbonate precipitation. However, based on fine‐scale textural relationships within the rock, we infer that the other microbialite textures were affected by diagenetic crystal growth, which reaffirms the importance of considering paragenetic history when describing and comparing ancient microbialites. The trace and rare earth element geochemistry of primary marine cements in the Callison Lake Formation microbialites suggests that the unit was deposited in a stratified, dominantly anoxic basin, with oxygenation above a very shallow chemocline. The relative depletion of chalcophile elements implies a euxinic depositional setting. This case study highlights the complexity of unlaminated microbialites and emphasises the need for detailed documentation of microbialites at multiple scales.

## 1 Introduction

Microbialites are carbonate rocks accreted as a result of benthic microbial activity (Burne and Moore 1987). Microbialite growth records the interaction of a microbial community with its environment, meaning that microbialite morphology is dependent on depositional conditions (e.g., Bosak et al. 2013), microbial community (e.g., Sumner et al. 2016) and seawater chemistry, in particular carbonate saturation state (e.g., Planavsky and Ginsburg 2009; Szilagyi et al. 2020). Understanding how these variables may have changed through time is important for reconstructing the co‐evolution of environments and microbial communities (e.g., Grotzinger and Knoll 1999).

One challenge in microbialite research is that broad terminology tends to mask the great variation and complexity observed in microbialites throughout geological time. This is a particular problem for the unlaminated forms of microbialite, including those described as thrombolites distinguished by their clotted mesostructural fabric (Shapiro 2000), dendrolites composed of small dendritic shrubs (Grey and Awramik 2020) and leiolites with no discernible fabric (Riding 2000). The defining characteristics of unlaminated microbialites (e.g., “clots”, “shrubs”, “calcimicrobes”) are broad and subjective, meaning that the same texture may be labelled with different names, particularly if the quality of preservation varies, and many textures lie within the acceptable definition of each category.

Despite the limitations in terminology, detailed descriptive case studies of unlaminated microbialites allow broad trends in microbial ecosystems to be discerned. In the earliest examples of Precambrian thrombolites, microscopic texture is characterized by a distinction between simple microcrystalline “clots” and coarser dolomite crystals (Kah and Grotzinger 1992; Tang et al. 2013; Dongjie et al. 2013; Nomchong and Van Kranendonk 2020). Tonian case studies suggest an increase in the preserved complexity of unlaminated microbialites, as microscale textures can be differentiated into categories such as diffuse or distinct microcrystalline clots, cyanobacterial calcimicrobes and cellular crusts (Turner et al. 1993; Harwood and Sumner 2011, 2012). Notably, the Tonian also includes the emergence of the first large‐scale unlaminated framework reefs (Turner et al. 1997). Later in the Neoproterozoic, thrombolites are more frequently recognized, particularly in association with Ediacaran macrofossils such as *Cloudina* (Hofmann and Mountjoy 2001; Amthor et al. 2003; Grotzinger and Al‐Rawahi 2014; Grotzinger et al. 2005; Warren et al. 2011, 2017). Finally, in the Phanerozoic, unlaminated microbialites are constructed by diverse calcimicrobes such as *Renalcis* and *Epiphyton*, which peak in abundance in the Cambrian and early Ordovician (e.g., Hong et al. 2012; Liu et al. 2017).

Overall, there is an apparent increase in the textural diversity of non‐stromatolitic microbialites through time. However, this trend remains speculative due to the sparsity of detailed case studies, and diagenesis and recrystallization likely obscure the primary textures in many ancient samples. To eventually understand the deep‐time record of unlaminated microbialites, we need additional, detailed studies which describe microbialites at multiple spatial scales and consider the paragenetic history of the rocks in question.

Here, we present new descriptions of and geochemical analyses of “clotted” microbialite textures of the ca. 745 Ma Callison Lake Formation in Yukon Territory, northwest Canada, which have been previously identified (Strauss et al. 2015) but not yet studied in detail. We document their occurrence as a reef framework as well as the mesoscale and microscale characteristics of these microbialites, adding to the existing record of Precambrian microbialite diversity. We also investigate the potential primary and secondary factors contributing to microbialite morphology. The redox conditions in which these microbialites formed are explored by examining the trace element and rare earth element compositions of primary marine cements that encrust microbialite frameworks.

## 2 Geological Background

### 2.1 Regional Geology

**FIGURE 1:**
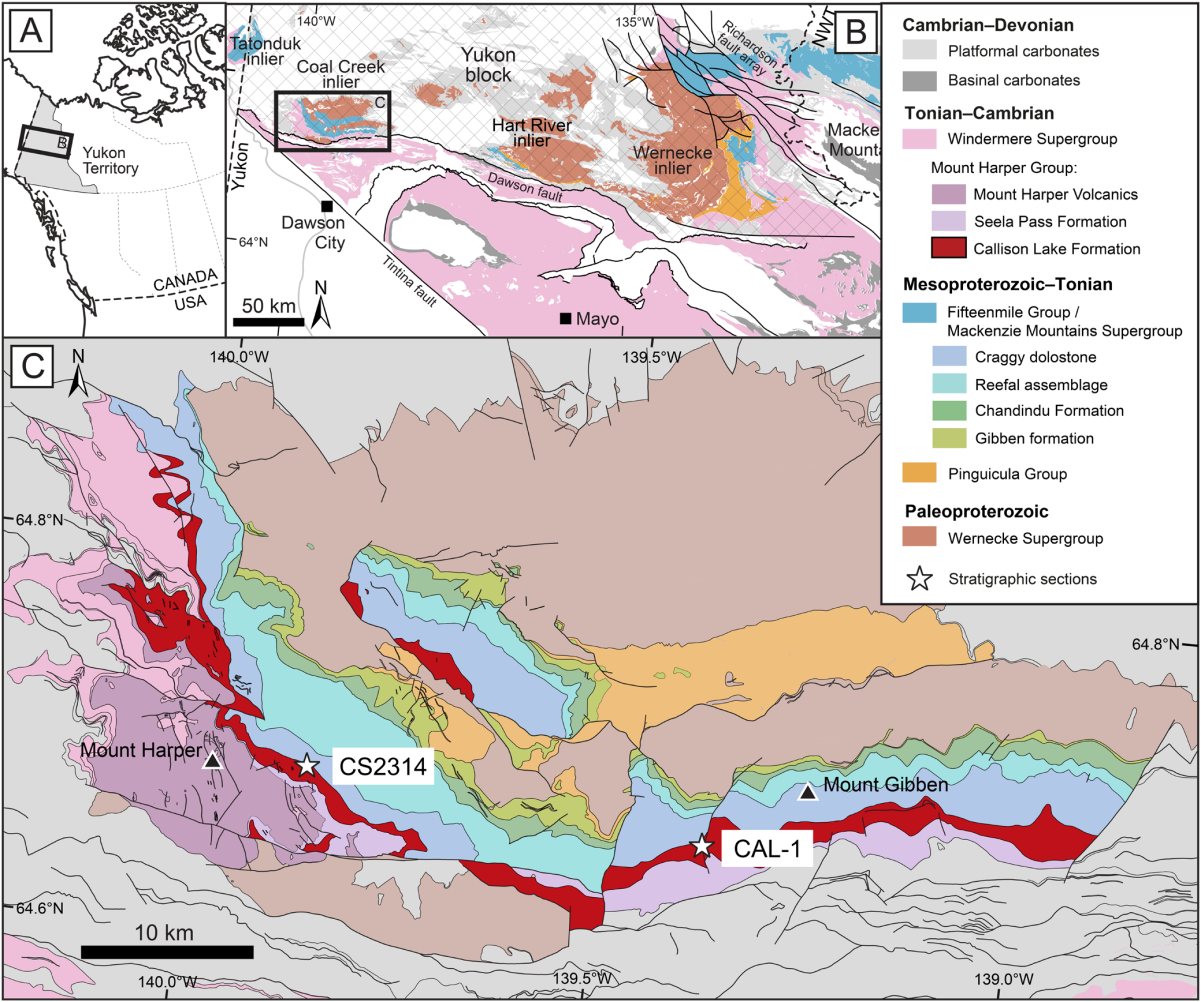
Bedrock geology of the study area. (A) Location of the study area with respect to northwest Canada. (B) Inset of (A); simplified bedrock geology of the Yukon, emphasizing Proterozoic Supergroups after Colpron et al. (2016). The location of Yukon block inliers has been annotated. (C) Inset of (B); geological map of the Coal Creek inlier after Strauss, Rooney, et al. (2014); Strauss, Roots, et al. (2014). Colours for the Wernecke Supergroup and Pinguicula Group are consistent with subfigure B. Tonian strata of the Fifteenmile Group and Windermere Supergroup are separated into their constituent formations.

**FIGURE 2:**
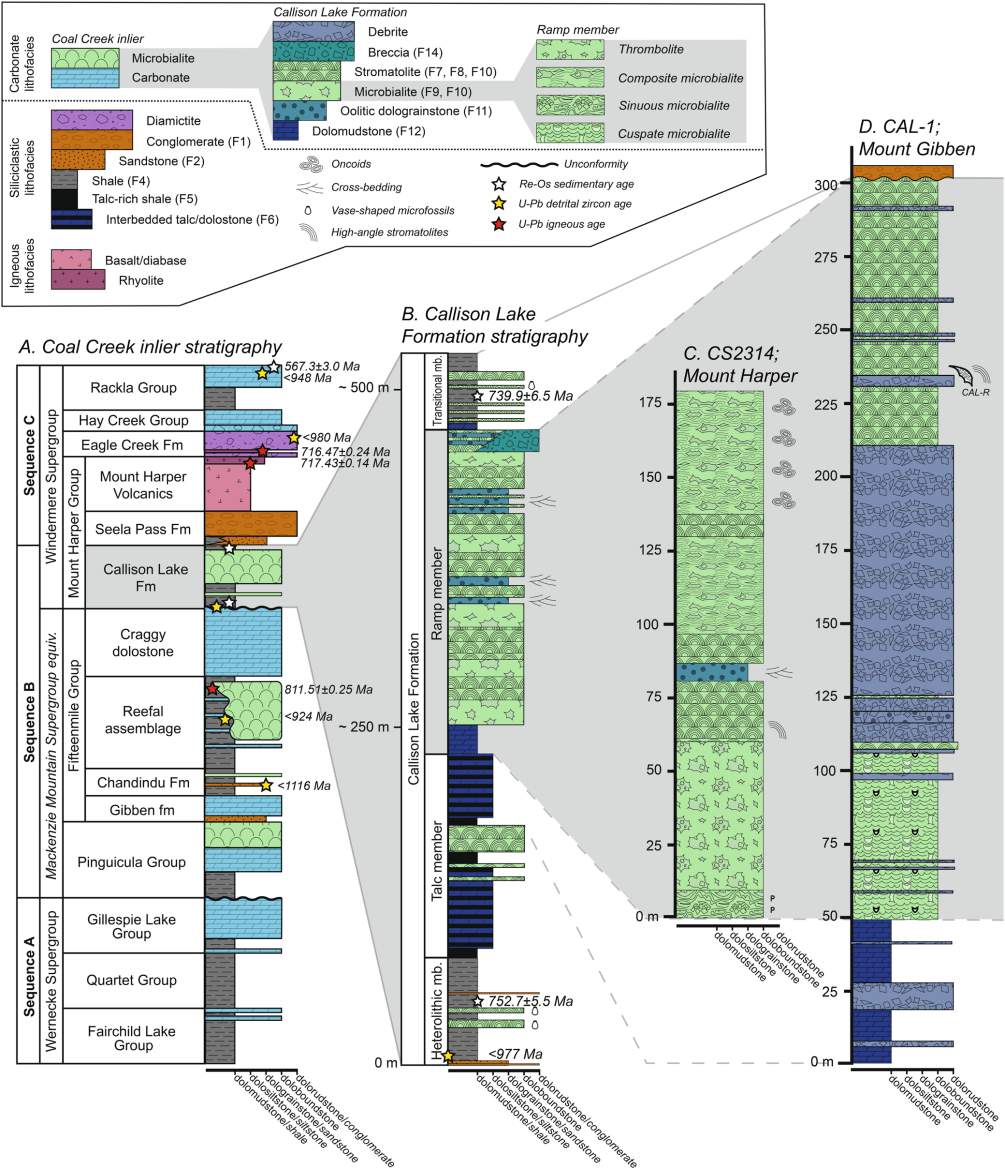
Stratigraphic logs of the Callison Lake Formation. (A) Regional stratigraphy of the Coal Creek inlier, adapted from figure 2 of Strauss et al. (2015) and updated based on recent work (Macdonald et al. 2012, 2018; Strauss et al. 2015; Furlanetto et al. 2016; Busch et al. 2021; Webb and Ambrose 2024; Spruzen et al. 2025). All radiometric ages are given in Ma (Macdonald, Schmitz, et al. 2010; Strauss, Rooney, et al. 2014; Strauss, Roots, et al. 2014; Rooney et al. 2015, 2020; Gibson et al. 2021). The ‘shale’ lithofacies encompasses siltstone in this log. (B) Summarized geology of the Callison Lake Formation, after member descriptions and figure 8 of Strauss et al. (2015). Lithofacies denoted by ‘(F_)’ are described in table 3 of Strauss et al. (2015), with the following minor adjustments: Stromatolite facies are grouped, intraclast breccia is incorporated into the microbialite/stromatolite facies, and minor facies (recrystallized dolomite, variegated siltstone/shale) are excluded. (C, D) Logs of the stratigraphic sections CS2314 and CAL‐1, for which the ‘microbialite’ lithofacies has been divided into four new microbialite lithofacies, described in this study.

The Yukon block is a crustal promontory on the northwest corner of Laurentia (Abbott 1997; Rainbird et al. 1996; Figure 1). Inliers of the Yukon block preserve thick successions of Proterozoic strata, which along with Proterozoic strata in the Northwest Territories and Victoria Island, are commonly subdivided into 3 unconformity‐bound “sequences” (Young et al. 1979; Rainbird et al. 1996; Figure 2). Basal Sequence A includes the polydeformed siliciclastic and carbonate rocks of the Wernecke Supergroup (Delaney 1986; Furlanetto et al. 2016), dated at ca. 1.6 Ga (Thorkelson et al. 2005; Furlanetto et al. 2013). The overlying Sequence B includes the Mesoproterozoic Pinguicula Group (Eisbacher 1981; Medig et al. 2010, 2012), the ca. 1000–800 Ma Fifteenmile Group (Roots and Thompson 1992; Macdonald and Roots 2010; Macdonald et al. 2012), and the lower Mount Harper Group whose basal unit is the Callison Lake Formation (Mustard and Roots 1997). The contact between the upper Fifteenmile Group and the Callison Lake Formation is a disconformity in the Coal Creek Inlier, but in the Hart River Inlier, it is a prominent angular unconformity, below which the upper Fifteenmile Group has been removed (Halverson et al. 2012; Strauss et al. 2015). Sequence C includes all Cryogenian and Ediacaran strata, as well as the ca. 717.5 Ma Mount Harper Volcanics, which underlie and provide a maximum age constraint for the basal Cryogenian glacial rocks of the Eagle Creek Formation in the Coal Creek Inlier (Macdonald, Smith, et al. 2010; Macdonald et al. 2018). Sequence C strata were buried by Phanerozoic passive margin sediments (Busch et al. 2021) and Proterozoic rocks were later exhumed by Mesozoic contraction (Mustard and Roots 1997). In the Coal Creek inlier, lower Sequence B rocks have been shown to have experienced only low‐grade metamorphic alteration indicated by partial sericitization of muds (Kunzmann et al. 2014).

In the Coal Creek inlier, the Callison Lake Formation is the basal unit of the Windermere Supergroup (Figure 2), which can be broadly correlated throughout the western Cordillera of North America and records multi‐stage rifting of the supercontinent Rodinia (ca. 780–700 and ca. 575–540 Ma) and the rift‐to‐drift transition of the western edge of Laurentia (Colpron et al. 2002; Busch et al. 2021). However, the Windermere Supergroup is expressed very differently across different sub‐basins. In the Coal Creek inlier, its basal unit is the Callison Lake Formation, which was deposited within localized transtensional basins that formed during early‐stage rifting of Rodinia (Strauss et al. 2015). The Callison Lake Formation is absent in the Wernecke Mountains of eastern Yukon, but given its age and tectonic context, it is considered correlative with the Coates Lake Group of the Mackenzie Mountains in the Northwest Territories, as well as Tonian strata in the “ChUMP” (Chuar, Uinta Mountains, and Pahrump Groups) basins of the southwest USA (Strauss, Rooney, et al. 2014; Strauss, Roots, et al. 2014; Strauss et al. 2015). Notably, the Callison Lake Formation is directly correlated with the Beck Spring Dolomite (California, USA) on the basis of the Islay carbon isotope excursion in the upper part of both units (Strauss, Rooney, et al. 2014; Strauss, Roots, et al. 2014; Smith et al. 2015) and the occurrence of vase‐shaped microfossils (Licari 1978; Strauss, Rooney, et al. 2014; Strauss, Roots, et al. 2014).

The 150‐ to 500 m‐thick Callison Lake Formation comprises four informally defined members spanning three transgressive‐regressive sequences (Strauss et al. 2015). The basal Heterolithic member includes siliciclastic strata deposited in a marginal marine setting at its base, followed by black shale and isolated reef bioherms that record transgression (Strauss et al. 2015). The overlying peritidal, evaporitic Talc member records a regressive phase and is followed sharply by the Ramp member, interpreted to record abrupt transgression and deposition on a carbonate ramp (Strauss et al. 2015). The upper part of the Ramp member and the succeeding Transitional member record syndepositional faulting, with asymmetrical basin fill geometries and abrupt lateral changes in facies (Strauss et al. 2015). Shales and conglomerates of the Seela Pass Formation disconformably overlie the Transitional member (Strauss et al. 2015).

The Callison Lake Formation has two depositional age constraints from Re‐Os dating of black shales in the Coal Creek inlier, with a 752.7 ± 5.5 Ma black shale horizon in the Heterolithic member (Rooney et al. 2015), and a 739.9 ± 6.1 Ma black shale horizon bearing vase‐shaped microfossils in the Transitional member (Strauss, Rooney, et al. 2014; Strauss, Roots, et al. 2014; Figure 2). Therefore, the Ramp member of the Callison Lake Formation—the focus of this study—was likely deposited ca. 745 Ma.

The Ramp member is up to ~400 m thick and includes diverse facies but consists predominantly of microbialite boundstones and coated grainstones. Given the subtidal, high‐energy facies associations, Strauss et al. (2015) proposed that the Ramp member represents deposition on a carbonate ramp with localized patch reefs and ooid shoals, although these authors note that it is possible the Ramp member represents a more substantial reef platform. In either case, the member records a deepening trend to the SSE, from peritidal‐ to subtidal‐dominated paleoenvironments (Strauss et al. 2015).

## 3 Methodology

### 3.1 Field Geology and Petrography

Two stratigraphic sections (Figure 2) were measured using the lithofacies described in Strauss et al. (2015). The Mount Harper section (CS2314; 64.696578° N, 139.858908° W) is within 100 m of section J1210 of Strauss et al. (2015), and its base corresponds to the base of the Ramp member. The Mount Gibben section (CAL‐1; 64.672717° N, 139.378117° W) is located approximately 20 km to the east, between sections J1218 and J1219 of Strauss et al. (2015). CAL‐1 can be correlated to the Ramp member based on the dominance of microbialite textures and the (unconformable) contact with the Seela Pass conglomerate at the top of the section (Figure 2). Given the scope of our study and the diversity of microbialites in the stratigraphic sections, we divided the “Microbialite (F9)” lithofacies of Strauss et al. (2015) into four microbialite facies (Figure 2).

Polished 30 μm thin sections of microbialite samples were analyzed with transmitted light microscopy, cathodoluminescent microscopy and Laser Ablation Inductively‐Coupled Plasma Mass Spectrometry (LA‐ICP‐MS). Petrographic analysis and description were done with a Zeiss Axio Scope A1 Polarizing Microscope. We adopt the terms used by Grey and Awramik (2020) in our field and petrographic descriptions, although we have made some minor adjustments and established additional terminology for the purpose of this manuscript. For clarity, all definitions for descriptive terms are provided in the supplement (Table S1, Figure S1).

A representative subset of polished thin section slides were carbon‐coated for Scanning Electron Microscopy (SEM) and Energy Dispersive Spectroscopy (EDS) analysis using a Hitachi SU5000 field emission SEM attached to an Oxford Silicon Drift Detector X‐MAX. Back‐Scattered Electron (BSE) and EDS images were taken at an accelerating voltage of 15 kV and a working distance of 11 mm. AZtec software (version 4.4) was used to process and classify EDS spectra and to generate EDS maps through image stitching.

Cathodoluminescent microscopy was carried out on a Nuclide ELM2B Cathodoluminoscope connected to a Wild M400 Photomacroscope, with operating conditions of 8 kV and a 0.6 mA beam current.

### 3.2 Geochemistry

Trace element concentrations, including rare earth elements, were determined on 100 μm petrographic sections cut from the same blocks as the thin sections. Analyses were performed using a Helex 193 nm ArF laser ablation system coupled to an Agilent 7700× quadrupole ICP‐MS, with a 5 Hz laser repetition rate, 9 mJ laser energy, and laser HV of 1.1 kV. Ablation spot size was 100 μm. The limit of detection for rare earth elements ranged from 0.3–2.2 ppb. Other trace element detection limits ranged from 1 to 50 ppb, except for Mn (390 ppb), Fe (240 ppb), and Cr (120 ppb). Any trace element concentrations less than three times the limit of detection were not used in our analysis.

The bracketing standard was NIST‐612 and external standards used were MACS3 and JCp‐1. Ca was used as an internal standard, assuming stoichiometric concentration for dolomite. In isopachous cements, suitable ablation spots were identified based on the preservation of primary growth banding on a scale of 10–50 μm, seen in both plane polarized light and cathodoluminescent petrography (Section 4.1). All LA‐ICP‐MS data are provided in (Table S3).

Rare earth element plus Yttrium (REE + Y) distribution patterns are normalized to Post‐Archean Average Shale (PAAS; McLennan 1989). Reported total REE concentrations do not include Y. Average geochemical data are reported as (mean ±1 standard deviation; median). Ce anomalies were calculated using Ce/Ce* = Ce_N_/(Pr_N_^2^/Nd_N_; Lawrence et al. 2006), Eu anomalies were calculated using Eu/Eu* = Eu_N_/(0.67*Sm_N_ + 0.33*Tb_N_; Bau and Dulski 1996), and the heavy‐to‐light rare earth element ratio was calculated using HREE/LREE = 0.2*(Ho_N_ + Er_N_ + Tm_N_ + Yb_N_ + Lu_N_)/0.25*(La_N_ + Ce_N_ + Pr_N_ + Nd_N_). Where appropriate, Ce and Eu anomalies from previous studies were recalculated with these formulae to facilitate comparison. As is convention, we refer to Ce/Ce* and Eu/Eu* anomalies as positive when greater than 1, and negative when less than 1.

## 4 Results

### 4.1 Microbialite Facies Descriptions

The Mount Harper and Mount Gibben stratigraphic sections record microbialite‐dominated carbonate framework fabrics in the Ramp member of the Callison Lake Formation (Figure 2). Microbialites include unlaminated microbialites and stromatolites, and these may also be intergrown. Stromatolitic facies tend to be recorded in the upper stratigraphy, particularly at Mount Gibben, where they may form steep growth fabrics. Unlaminated microbialites are divided into four different facies including thrombolites, composite microbialites, sinuous microbialites and cuspate microbialites (Figure 2). At Mt. Harper, these unlaminated microbialites dominate the section and may have fenestral fabrics, occurring with ooids and oncoids (Figures 2 and S2). At Mount Gibben, the unlaminated microbialites are best developed towards the base of the section and are interbedded with carbonate breccias and debrites (Figure 2).

#### 4.1.1 Thrombolite

The thrombolite facies is defined by cavity‐dense, cement‐lined clotted microbialite and is found in the western Mount Harper section. In this section, the thrombolite occurs as poorly exposed subcrop, but the section is adjacent to a large cliff with continuous exposure. The thrombolite passes upward into ~30 m of micritic stromatolites forming decimeter‐scale domes, with an interval of cross‐bedded oolitic grainstone.

**FIGURE 3:**
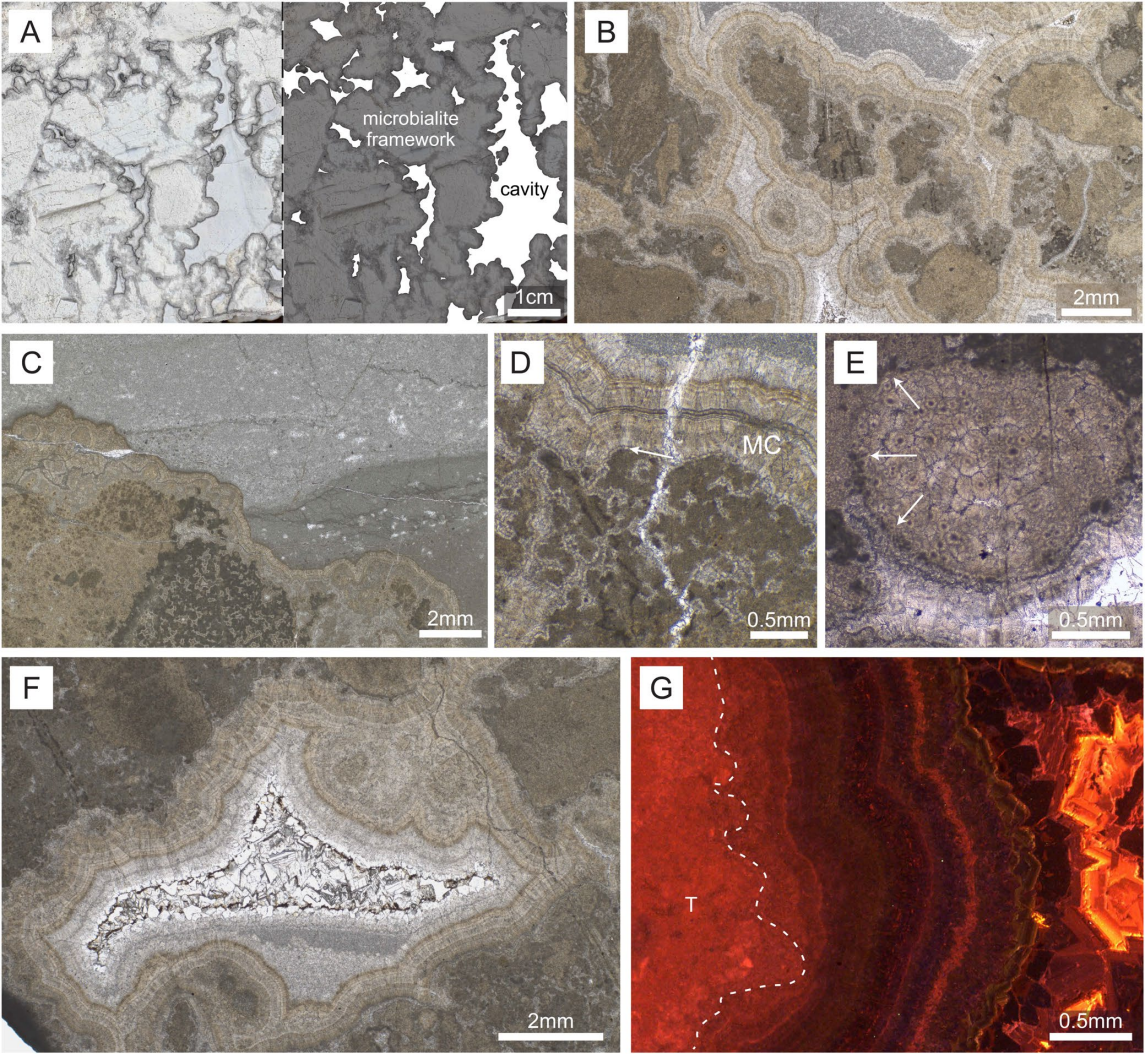
Mesostructure and microstructure of the Callison Lake Formation thrombolite. (A) Outcrop photograph of the thrombolite facies (left), with microbialite framework outlined in black and cavities coloured in white for clarity (right). (B) Plane‐polarized light petrographic image of a typical thrombolite texture. (C) Plane‐polarized light petrographic image of thrombolite texture, focusing on a large cavity filled with laminated dolomicrite. (D) Plane‐polarized light petrographic image of the texture within a mesoclot, with isopachous fibrous marine cement (MC) coating the thrombolite. Arrow points to a microclot coated with a thin rim of cement. (E) Plane‐polarized light petrographic image of interlocking microclot texture, which has a sharp boundary (arrows) with the adjacent uniform microcrystalline dolomite. (F) Plane‐polarized light petrographic image of a geopetal structure, shown by internal sediment overlaying marine cement but only partially filling a cavity, with the rest filled with late‐stage cement. (G) Cathodoluminescent petrographic image of marine cements growing from the thrombolite (T) within a cavity. Note the colour banding within the isopachous cements, and the contrast with the adjacent (right hand side) rhombic late‐stage dolomite with bright yellow growth zonation.

In outcrop, the microbial framework of the thrombolite facies has no clear growth direction or orientation (Figure 3A). The framework is characterized by abundant cavities filled with light dolomicrite (Figure 3A). At the edges of cavities, cement outlines distinct mesoclots 3–10 mm in diameter (Figure 3A). These mesoclots protrude away from the dense microbialite framework into the cavity, and some appear as isolated ‘clouds’ within the cavity, resulting from a two‐dimensional cut through a three‐dimensional framework. Close to cavity walls, bright white microcrystalline dolomite forms distinct mesoclots approximately 0.5–2 mm across. Further from the cavities, the microbialite is uniform and microcrystalline (Figure 3A). All components consist of dolomite, with no calcite being present, and SEM‐EDS mapping shows no variation in the major element composition of dolomite. We note that the mesostructure of the Callison Lake Formation thrombolite is strongly similar to the thrombolites of the correlative Beck Spring Dolomite in the Death Valley region (Harwood and Sumner 2011, note figures 3E, 7D, 8A–F, 9), as well as cored sections of “shrub microbialite” in the ca. 760 Ma Devede Formation in northwestern Namibia (Hood et al. 2015, note figure 10E–H).

In thin section, the cavities in the Callison Lake thrombolite are coated with fibrous isopachous, radial and fascicular length‐slow dolomite cements (Figure 3B–G; Section 4.2) and partially filled with laminated dolomicrite (Figure 3C). Within the microbialite, thrombolite mesoclots are polylobate (Figure 3B) and are comprised of two end‐member textures. The first is rounded distinct microclots, which are 100–150 μm in diameter and often clustered together (Figure 3D). These microclots are dark in plane‐polarized light, and at high magnification, they consist of very fine‐grained (< 10 μm) crystals of cloudy, inclusion‐rich dolomite and a small amount of an opaque phase. The opaque material is bright in reflected light with a white card, consistent with organic inclusions (Figure S3; Folk 1987). Similarly, in SEM‐EDS a phase can be identified with low atomic weight (Figure S3), but its elemental composition has no clear distinction from the dolomite groundmass; this phase may also be attributed to possible organic matter. Close to the edges of internal framework cavities, clusters of microclots tend to be very clearly defined by a rim of thin (~40 μm) dolomite cement. Away from the cavity boundaries, the texture grades to uniform microcrystalline dolomite, and distinct microclots are no longer distinguishable, although at high magnification the fabric composition is the same as that of the microclots.

The second endmember texture comprises round, mutually interfering areas of microcrystalline dolomite (0.03–0.10 mm diameter) with dark, diffuse centres (0.01–0.03 mm) and clear rims (Figure 3E). The fabric is microcrystalline throughout; the distinction between the cores and rims is in colour rather than crystal size. At high magnification, the dark centres have a similar composition to the fabric of the distinct microclots. In reflective light the centres are lighter than the rims. We refer to this texture as ‘interlocking microclots’.

While distinct microclots and interlocking microclots represent the two textural endmembers, much of the thrombolite texture lies on a spectrum between the two, comprised of a variable proportion of cement and dark microcrystalline dolomite. The contacts between distinct microclots, uniform microcrystalline dolomite, and interlocking microclots may be gradational, or the interlocking microclots may be present as distinct, round zones 0.5–1.5 mm in diameter with sharp boundaries (arrows in Figure 3E).

#### 4.1.2 Composite Microbialite

**FIGURE 4:**
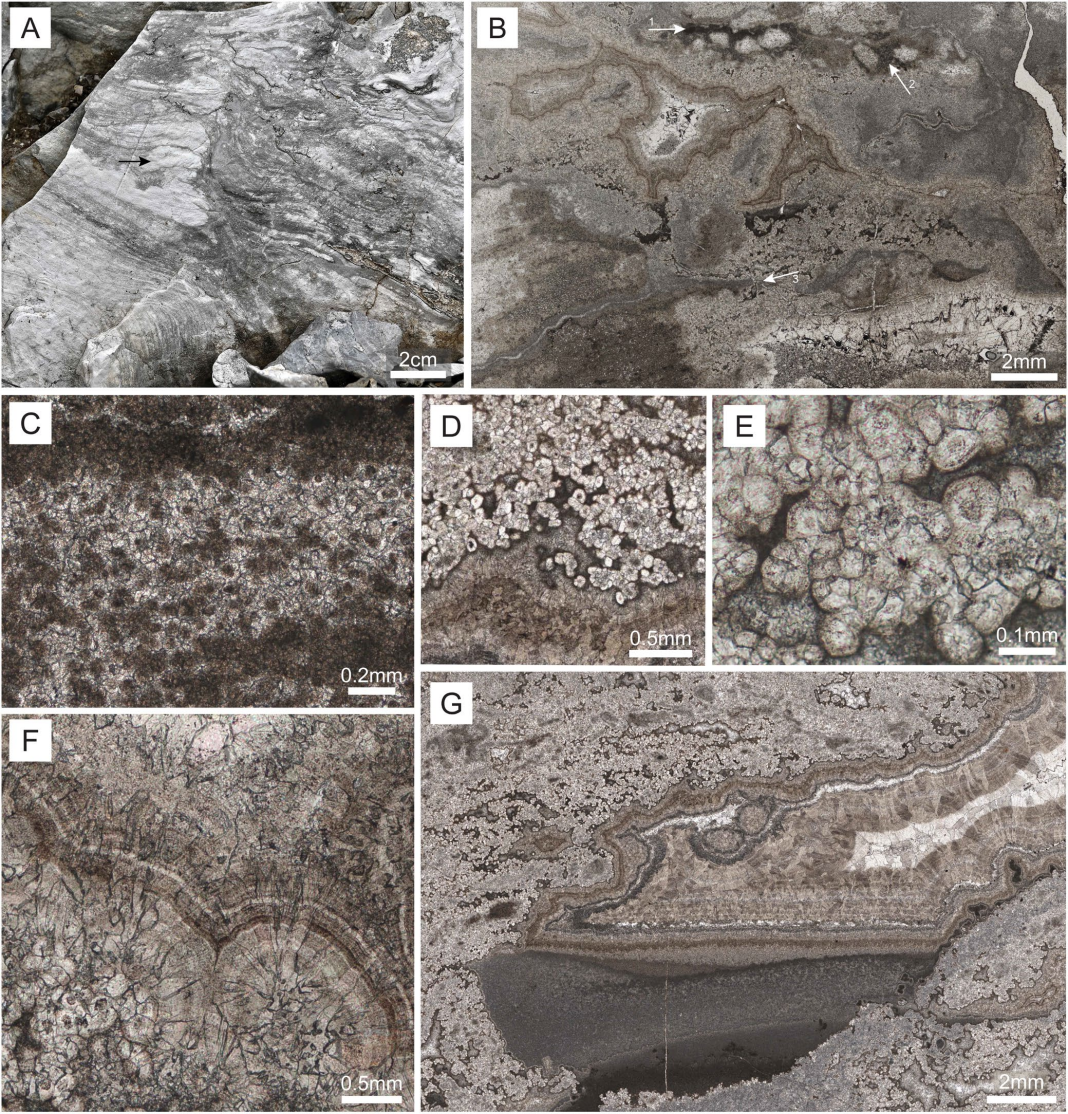
Mesostructure and microstructure of the Callison Lake Formation composite microbialite. (A) Outcrop photograph of the composite microbialite. Arrow points to an area of unlaminated microcrystalline dolomite; stromatolite laminae bend around this area on the right‐hand side. (B) Plane‐polarized light petrographic image of the composite microbialite. Arrows 1 and 2 point to bands of dark dolomicrite. Arrow 3 highlights where uppermost stromatolite laminae continue across an area of unlaminated sparry microclots. (C) Plane‐polarized light petrographic image of diffuse microclots which form stromatolitic laminae. (D) Plane‐polarized light petrographic image of sparry microclot framework above primary cements infilling a cavity. (E) Plane‐polarized light petrographic image of sparry microclot texture, showing organic‐rich interiors with subhedral sparry exteriors. (F) Plane‐polarized light petrographic image of growth banding within primary marine cements lining a cavity. (G) Plane‐polarized light petrographic image of cavity partially filled with internal sediment, lined with marine cements.

Above the cross‐bedded oolitic grainstone in the Mount Harper section is an interval of intergrown stromatolite and pale unlaminated microbialite, which we designate the composite microbialite facies. In outcrop, the stromatolites of the composite microbialite facies variably display truncation and high‐angle laminae to recumbent lamination (Figure 4A). Laminae are defined by laterally discontinuous, mm‐scale alternations of white and grey microcrystalline dolomite. Cement‐lined cavities are elongate parallel to stromatolitic lamination. Stromatolite laminae also bend around distinct zones of pale uniform microcrystalline dolomite (Figure 4A), with similar texture in outcrop to the framework of the thrombolite facies. This unlaminated microbialite also has cavities, whose boundaries are formed by rounded protrusions from the surrounding microbialite. Scour surfaces and brecciation are common in the composite microbialite facies, and cements are ubiquitous, outlining the edges of cavities (Figure 4B) and brecciated microbialite. In the upper 30 m of the section, microbialites are associated with oncoids, which are 1–4 cm in diameter and distributed within a micritic matrix. Oncoids may include asymmetric partial‐dissolution textures (Figure S2D).

In thin section, the microbialite cavities are coated by isopachous, fibrous, length‐slow dolomite cements (Figure 4B,F,G). The microbialite framework itself displays a complex mosaic of textures (Figure 4B). The first texture is microclotted stromatolite (Figure 4B,C), in which laminae are defined by brown‐tinged microcrystalline dolomite, which may form streaky dark bands or layers of dense, diffuse microclots of 50–100 μm diameter (Figure 4C). Microspar crystal boundaries cut across these diffuse microclots.

We have defined the second texture as ‘sparry microclots’, comprised of 60–140 μm diameter sparry crystals with dark, microcrystalline centres (Figure 4B,D,E). These are round in shape but organized into a clotted framework, while boundaries between crystals are polygonal forming an approximately hexagonal pattern (Figure 4E). Between crystals, dark grey microcrystalline dolomite may coat the base of cavities as a geopetal structure, or coat the crystals equally regardless of orientation. The arrangement of the sparry microclots can be compared to the distinct microclot texture of the thrombolite facies, which has similar cavities, clots, and protrusions. In cross‐polarized light, crystals display uniform extinction.

The third texture is dominated by grey microcrystalline dolomite, which often has a gradational contact with the sparry microclots. The texture also includes some very dark isolated bands and patches (Figure 4B).

Stromatolitic and unlaminated textures are found in distinct zones within the bulk microbialite (Figure 4B). Boundaries are defined by a change in texture, which is sharp for the edge of stromatolite zones. At a smaller scale, the contacts between textures are variably sharp or gradational. In places, the uppermost stromatolite laminae continue across the unlaminated microbialite (Figure 4B), and sparry microclots are organised in elongated zones within stromatolite lamination, suggesting a continuum between the laminated and unlaminated textures.

#### 4.1.3 Sinuous Microbialite

**FIGURE 5:**
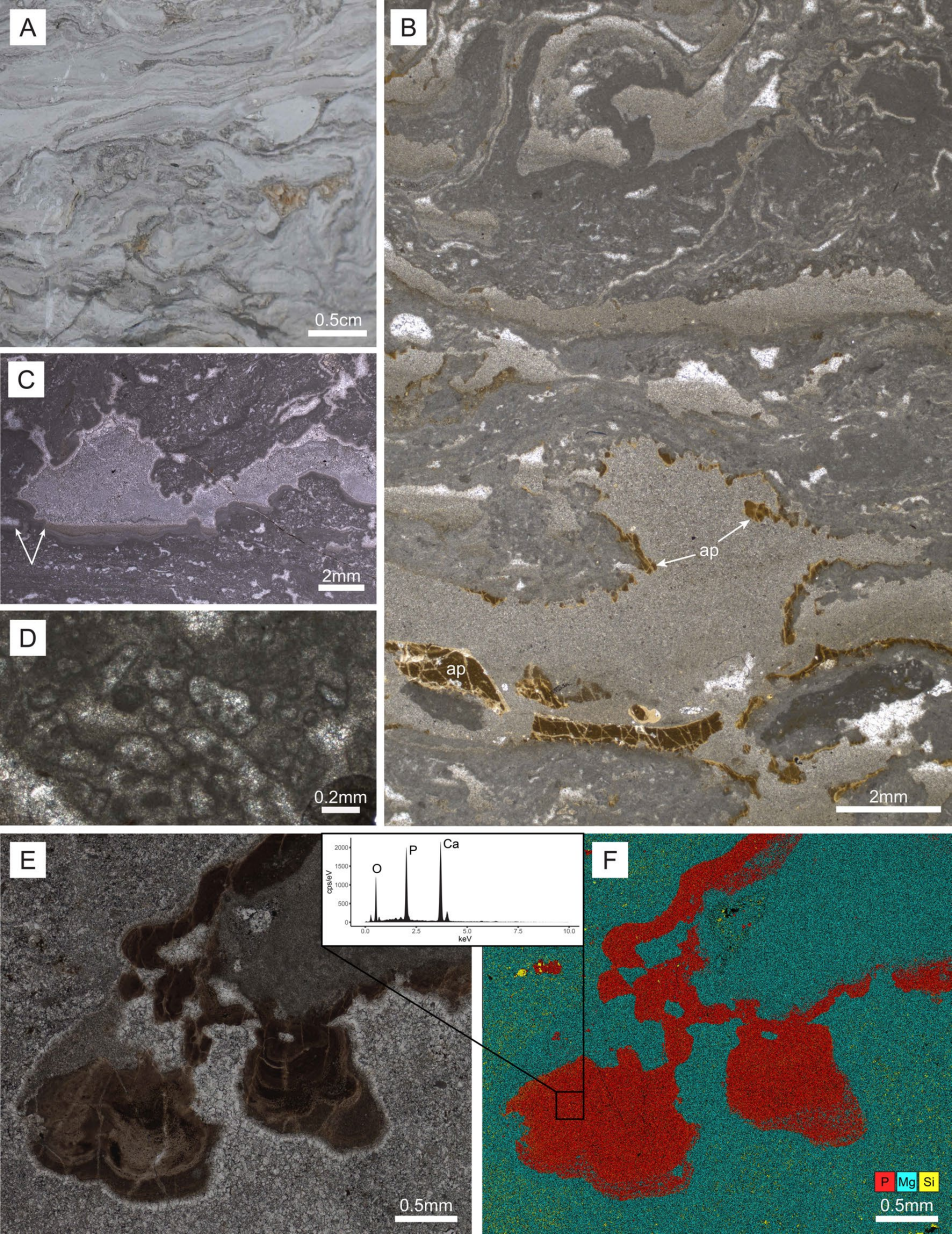
Mesostructure and microstructure of the Callison Lake Formation sinuous microbialite. (A) Outcrop photograph of the sinuous microbialite, specifically the complex, convolute cavities. (B) Plane‐polarized light petrographic image of sinuous microbialite with dark brown apatite (ap) adjacent to cavities. (C) Plane‐polarized light petrographic image of a cavity within the sinuous microbialite. Arrows point to internal sediment continuing across a bridge of microbialite in the centre of the cavity. (D) Plane‐polarized light petrographic image of cortoidal groundmass texture within the sinuous microbialite. (E) Plane‐polarized light petrographic image of an apatitic protrusion within the sinuous microbialite, adjacent to a cavity filled with internal sediment. (F) SEM‐EDS image of (E) with major element spectrum shown.

At the base of the Ramp member in the western section is a 10 m‐thick outcrop of laterally discontinuous breccia, fenestral dolosiltite, and microbialite. Breccia clasts are predominantly micritic, angular and tabular, between 5 and 30 mm long, and surrounded by a laminated dolosiltite matrix with local cross‐lamination. Rare rounded clasts are pale with polygonal cracks. Some fenestrae are elongate and aligned with bedding, and in thin section are seen to be separated by thin layers of cement. The microbialite is distinguished from the adjacent dolosiltite and dolomudstone based on complex sub‐vertical lamination and sinuous, irregular cavity shapes. Cavities are defined by the edges of polylobate microbialite, and elongate cavities are not always bedding‐parallel (Figure 5A).

In thin section, geopetal‐forming dolomicrite is common within microbial cavities (Figure 5B,C). Unlike the thrombolite and composite microbialite facies, larger cavities in this microbialite generally have planar bases with lobate upper surfaces, often with pendant structures protruding downwards (Figure 5B,C). Cavities are also rarely coated with isopachous cements. The microbialite itself is mostly comprised of dense microcrystalline dolomite peppered with abundant small cavities (Figure 5B), and in places, the texture grades into cortoids (micrite‐coated grains; Figure 5D), which are rounded, sometimes polylobate, and approximately 0.2–0.5 mm in diameter.

Apatite occurs at the margins of the microbialite close to sinuous cavities, preferentially taking the form of pendant bulbs (Figure 5B,E). Rare tabular‐shaped phosphatic layers occur at the base of larger cavities. These tabular layers cap a lower level of dolomicrite and are partially brecciated (Figure 5B).

The sinuous microbialite bears many similarities to the composite microbialite facies, including lateral variability, complex recumbent lamination, and alternation between laminated and unlaminated texture. However, the sinuous microbialite is assigned to a separate facies given that it has a starkly different small‐scale texture, which lacks microclots and includes cortoids, complex sinuous cavities, and apatite.

#### 4.1.4 Cuspate Microbialites

**FIGURE 6:**
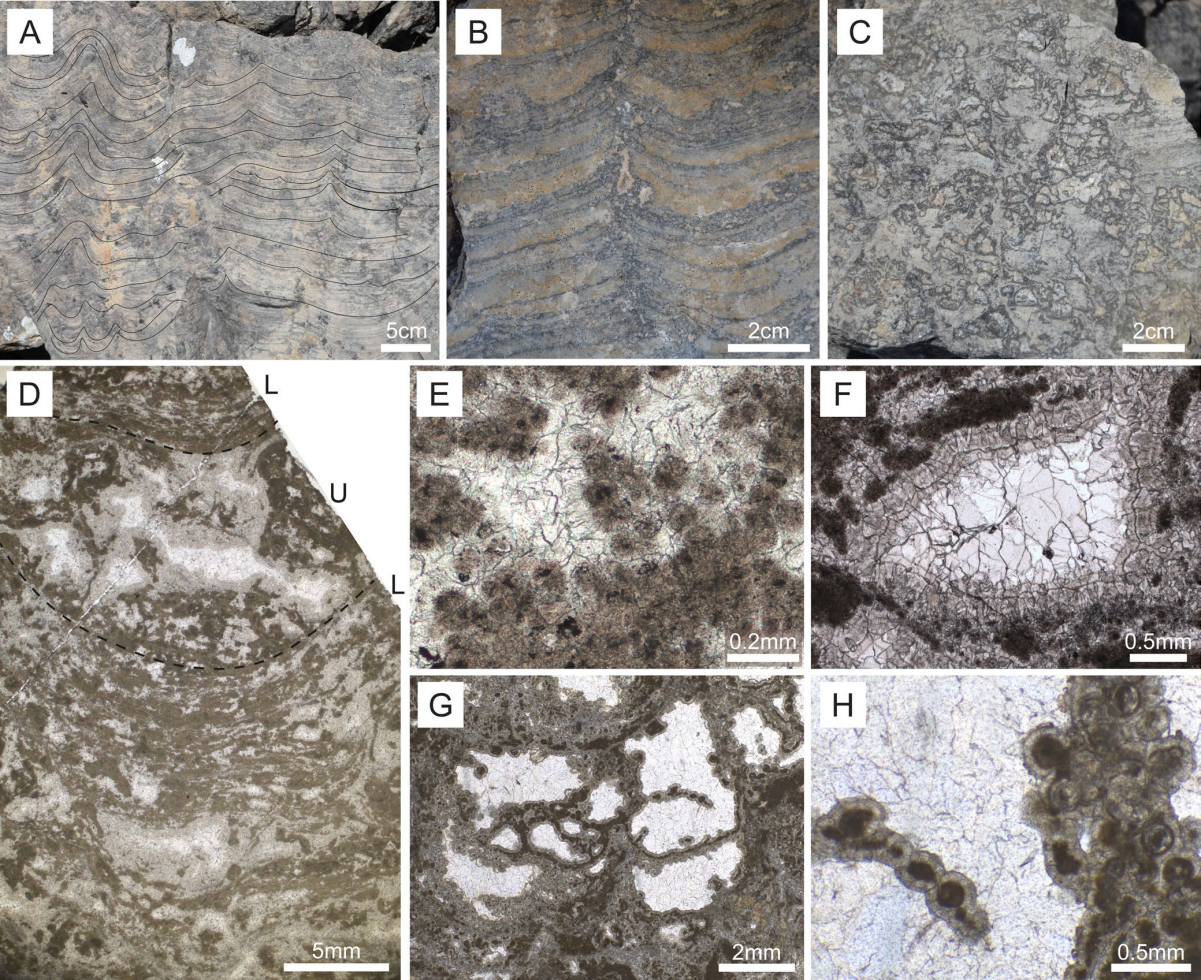
Microstructure of the Callison Lake Formation cuspate microbialite. (A) Outcrop photograph of a cuspate stromatolite, with laminations outlined in black. An unannotated version of the image is provided in the Supplement (Figure S2E). (B) Outcrop photograph of the centre of a stromatolite cusp, showing fenestrae filled with late‐stage spar. (C) Outcrop photograph of the cuspate microbialite facies, showing a local expression of cavity‐dense microbialite with no lamination and no clear growth direction. (D) Plane‐polarized light petrographic image of cuspate microbialite texture, showing laminated (L) and unlaminated (U) subsections, both of which contain cement‐lined cavities. (E) Plane‐polarized light petrographic image of diffuse microclots within the cuspate microbialite. (F) Plane‐polarized light petrographic image of a cement‐lined cavity within a laminated cuspate microbialite. (G) Plane‐polarized light petrographic image of cuspate chambered structures within sample CAL‐R (see Figure 2D for stratigraphic position). (H) Plane‐polarized light petrographic image of small‐scale texture of microclots comprising the cavity walls within cuspate chambered structures.

Cuspate microbialites are present in the Ramp member of the Mount Gibben area. The base of the section is ~50 m of fine‐grained, bedded, muddy dolostone with chert nodules, and thin beds of intraclastic breccia. Intraclasts are angular and include laminated microbialite fragments (Figure S2F). Fine‐grained facies pass upward into ~55 m of gray to dark gray non‐bedded massive dolostone with cuspate, scalloped stromatolites and cavities, which we designate the cuspate microbialite facies. In outcrop, cuspate laminae are stacked, smooth and concave‐upwards, with 5–10 cm spacing between cusps. The cusps mostly form sharp points, but in some instances transition to domed morphologies (Figure 6A). Cavities are common within the cuspate microbialites and are concentrated in the axes of cusps (Figure 6B). This facies is laterally discontinuous at the outcrop scale, and locally the texture may be dominated by cement‐lined cavities with no clear lamination or growth direction (Figure 6C). Overall, the cuspate microbialites presented in this work have similar mesoscale morphologies to the “tented” fenestrate microbialites of Sumner (2000) and other Precambrian cuspate stromatolites (O'Connell et al. 2022 and references within).

In thin section, the cuspate microbialite texture is dominantly laminated but has irregular gradations between laminated and unlaminated areas, both of which include cement‐lined cavities (Figure 6D). Cavity‐dense unlaminated microbialite may form a thick layer between stromatolite laminae (Figure 6D) or may be concentrated between cusps (Figure 6B). The small‐scale texture of the cuspate microbialite is the same in laminated and unlaminated areas, comprised of rounded diffuse microclots (Figure 6E). These microclots are similar in size (0.050–0.070 mm) and texture to the diffuse microclots in the composite microbialite facies. Where present, lamination results from alternation between layers dominated by microclots and cement (Figure 6D).

Stratigraphically above the cuspate microbialite facies in the Mount Gibben section is ~100 m of intraclastic to massive breccia (some marine cemented) with intervals of laminated stromatolites and rarer cuspate stromatolites. This is followed by ~30 m of low‐angle, m‐scale stromatolite mounds (sometimes silicified) and intraclastic breccias. Some metre‐scale stromatolite growth surfaces are sub‐vertical toward the N‐NE (ca. ~230–240 m in CAL‐1), and we identified small, 5 mm‐scale chambered structures growing from one of these vertically‐oriented surfaces. In petrographic thin section, chamber walls are 0.10–0.20 mm wide, and chambers are filled with blocky quartz spar (Figure 6G). Both chamber walls and dense microbialite are constructed by either light grey microcrystalline dolomite or microclots (Figure 6G). These cuspate microclots, at 0.1–0.25 mm diameter, are larger than the thrombolite distinct microclots and the composite microbialite sparry microclots. They consist either entirely of dark microcrystalline dolomite or of concentric dark layers (Figure 6H). Many microclots are coated with a thin rim of cement around 50 μm thick (Figure 6H).

### 4.2 Cement Petrography

Primary growth cavities within the thrombolite, composite microbialite and cuspate microbialite facies of the Callison Lake Formation are partially filled by fibrous isopachous, radial and fascicular length‐slow dolomite cements. These cements are commonly overlain by dolomicrite, which is laminated and contains clasts (0.1–1.5 mm diameter) in large cavities (Figure 3C). The dolomicrite often partially fills cavities, forming geopetal structures (Figure 3F). In the thrombolite and composite microbialite facies, cements are generally 0.5–1.5 mm thick and in plane‐polarized light, they display well‐preserved growth zonation characterized by colour changes on the order of 10–30 μm (Figures 3D,G and 4F). In cathodoluminescence, this zonation is characterised by alternation between dull purple, green and red luminescence, which is distinct from the bright red‐yellow growth zones of the rhombic, cavity‐occluding dolomite cements (Figure 3G).

In the thrombolite facies, fibrous dolomite cements are consistently overlain by laminated dolomicrite (Figure 3C,F), whereas laminated dolomicrite in the composite microbialite facies variably predates (Figure 4G) and postdates (Figure 4B) the fibrous cements. In some examples, composite microbialite cavities have a thin (~100 μm) rim of early fascicular length‐fast dolomite cement directly overlying microbialite and laminated dolomicrite.

Within the cuspate microbialite, cavities are lined with 0.2–0.4 mm‐thick fibrous fascicular slow dolomite cements (Figure 6F). The fine‐scale zonation in these cements is more variable than the thrombolite or composite microbialite facies, with some examples only showing one or two distinct growth zones.

### 4.3 Trace and Rare Earth Element Geochemistry

#### 4.3.1 Thorium, Zirconium, and Total Rare Earth Elements

The concentrations of Th, total REE (ΣREE) and Zr are typically used to screen for terrestrial contamination in component‐specific analysis of carbonates (Webb and Kamber 2000; Stacey et al. 2023). All components analysed from the Callison Lake Formation have low concentrations of Th, ΣREE and Zr, with the lowest concentrations in the isopachous cements (Figure S4). All measurements of cement Y/Ho are greater than 27, the Y/Ho ratio of PAAS (McLennan 1989; Figure S5).

#### 4.3.2 Trace Elements

**FIGURE 7:**
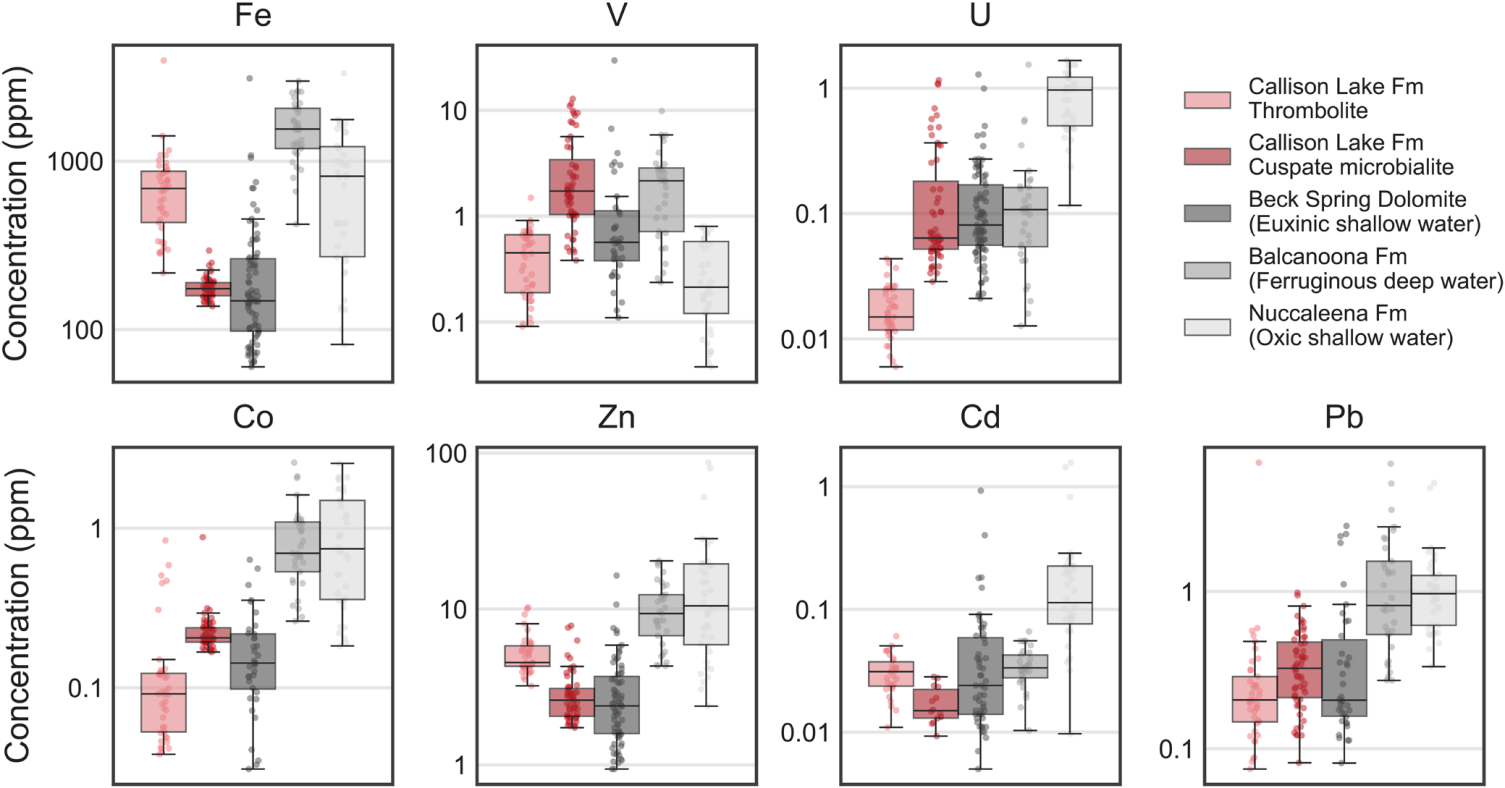
Boxplots of trace element LA‐ICP‐MS analyses of isopachous marine cements in the thrombolite facies and cuspate microbialite facies of the Callison Lake Formation. The data from our study is compared to other marine cement trace element data from the Tonian Beck Spring Dolomite (California, USA; Shuster et al. 2018) the Cryogenian Balcanoona Formation (South Australia; Hood and Wallace 2015) and the Ediacaran Nuccaleena Formation (South Australia; Lamothe et al. 2024). Beck Spring Dolomite data is from marine cements in all shallow marine facies. Balcanoona Formation data is from marine cements in deep water only, which is interpreted to be a ferruginous setting (Hood and Wallace 2014, 2015). Nuccaleena Formation data is from shallow environments only, which are interpreted to be an oxic setting (Lamothe et al. 2024).

In the thrombolite facies of the Callison Lake Formation, isopachous cements contain low concentrations of the redox‐sensitive elements V (mean 0.46 ± 0.30; median 0.45 ppm) and U (mean 0.018 ± 0.009; median 0.015 ppm). Within cuspate microbialite cavities, isopachous cements are slightly higher in V (mean 3.1 ± 3.3; median 1.7 ppm) and U (mean 0.20 ± 0.27; median 0.064 ppm) but still low (Figure 7). V and U also strongly correlate in the cuspate facies (*R* ^2^ = 0.84), but not the thrombolite facies (*R* ^2^ = 0.02; Figure S6). Isopachous cements within both microbialite facies have similar Mn concentrations, while Fe is much higher in the thrombolite facies (Figures 7 and S6). In the cuspate microbialite facies, Fe and Mn positively correlate (*R* ^2^ = 0.56; Figure S6), while in the thrombolite facies, Fe and Mn only display weak correlation (*R* ^2^ = 0.26; Figure S6).

Chalcophile elements have low concentrations within isopachous cements in both the thrombolite (Cd: 0.031 ± 0.011 ppm; Cu: 0.77 ± 0.84 ppm; Zn: 5.3 ± 1.6 ppm; Pb: 0.4 ± 1.0 ppm) and cuspate microbialite facies (Cd: 0.017 ± 0.006 ppm; Cu: 0.44 ± 0.42 ppm; Zn: 2.9 ± 1.3 ppm; Pb: 0.36 ± 0.21 ppm; Figure 7). These elements are strongly positively correlated in both facies (Figure S6), with the exception of Pb in the cuspate microbialite, which has zero to weakly negative correlation with Co, Cd, Cu and Zn. In the cuspate facies, Cd and Zn strongly positively correlate with the redox‐sensitive elements U and V, although Cd also correlates with Th, ΣREE and Zr, which are considered proxies for terrestrial contamination (Figure S6).

#### 4.3.3 Rare Earth Elements

**FIGURE 8:**
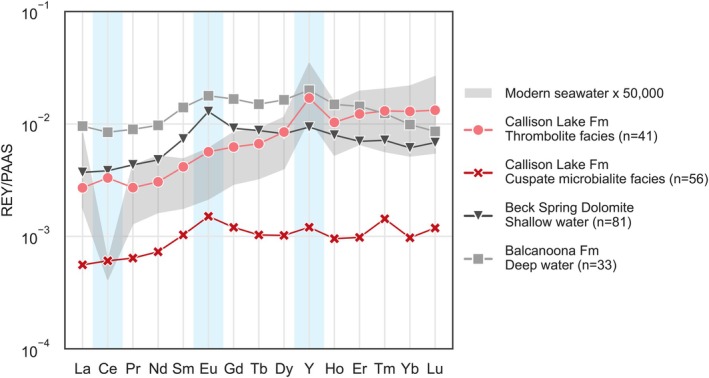
Average rare earth element plus yttrium (REE + Y) distribution patterns for marine cements, normalized to post‐Archean average shale (McLennan 1989). Data is from isopachous marine cements in the Callison Lake Formation thrombolite and cuspate microbialite facies. Comparative REE + Y distribution patterns are plotted for shallow environments from the Beck Spring Dolomite (Shuster et al. 2018) and deep water from the Balcanoona Formation (Hood and Wallace 2015). The grey background trace denotes 50,000 times REE + Y concentrations from modern Pacific seawater at a range of depths (Kamber 2010).

In the Callison Lake Formation, the PAAS‐normalized REE + Y distribution patterns of isopachous cements have the same shape as sedimentary components (Figure S7). These distribution patterns are distinct between the thrombolite facies and the cuspate facies (Figure 8). In the thrombolite marine cements, the normalized distribution pattern is similar to that of modern seawater in terms of its HREE enrichment, which results in a positive slope (Figure 8). This result is also reflected in the heavy to light REE ratio. Isopachous cements in the thrombolite facies have an elevated mean HREE/LREE of 4.5 ± 1.4 (median 4.5), whereas their counterparts in the cuspate microbialite facies have a mean HREE/LREE of 1.8 ± 0.4 (median 1.7). The Y/Ho ratios are also similar to modern seawater in the thrombolite facies, as cements have a Y/Ho mean of 45.5 ± 6.9 ppm (median 44.9 ppm), and all values fall within the range of modern seawater (36 and higher; Bau et al. 1997; Censi et al. 2007; Figure S5).

**FIGURE 9:**
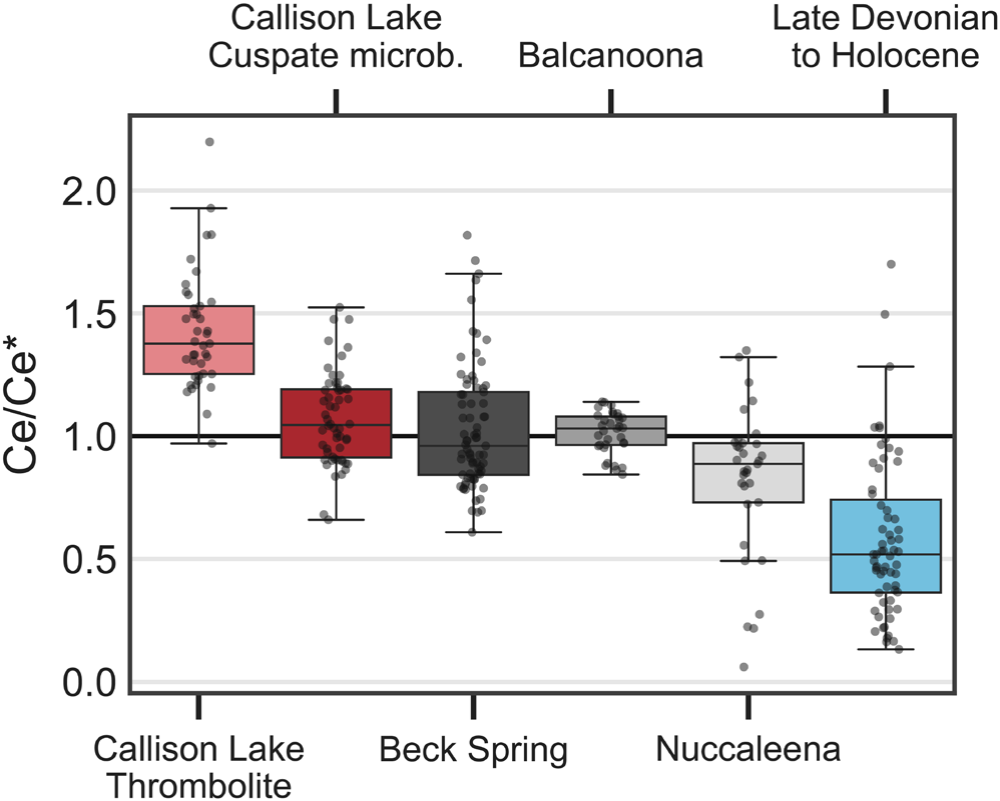
Ce/Ce* anomalies recorded in isopachous marine cements from the Callison Lake Formation thrombolite and cuspate microbialite facies. Comparative data is provided for marine cements from the Tonian Beck Spring Dolomite (Shuster et al. 2018), the Cryogenian Balcanoona Formation (Hood and Wallace 2015), the Ediacaran Nuccaleena Formation (Lamothe et al. 2024), and various strata from the Late Devonian onwards (Wallace et al. 2017).

**FIGURE 10:**
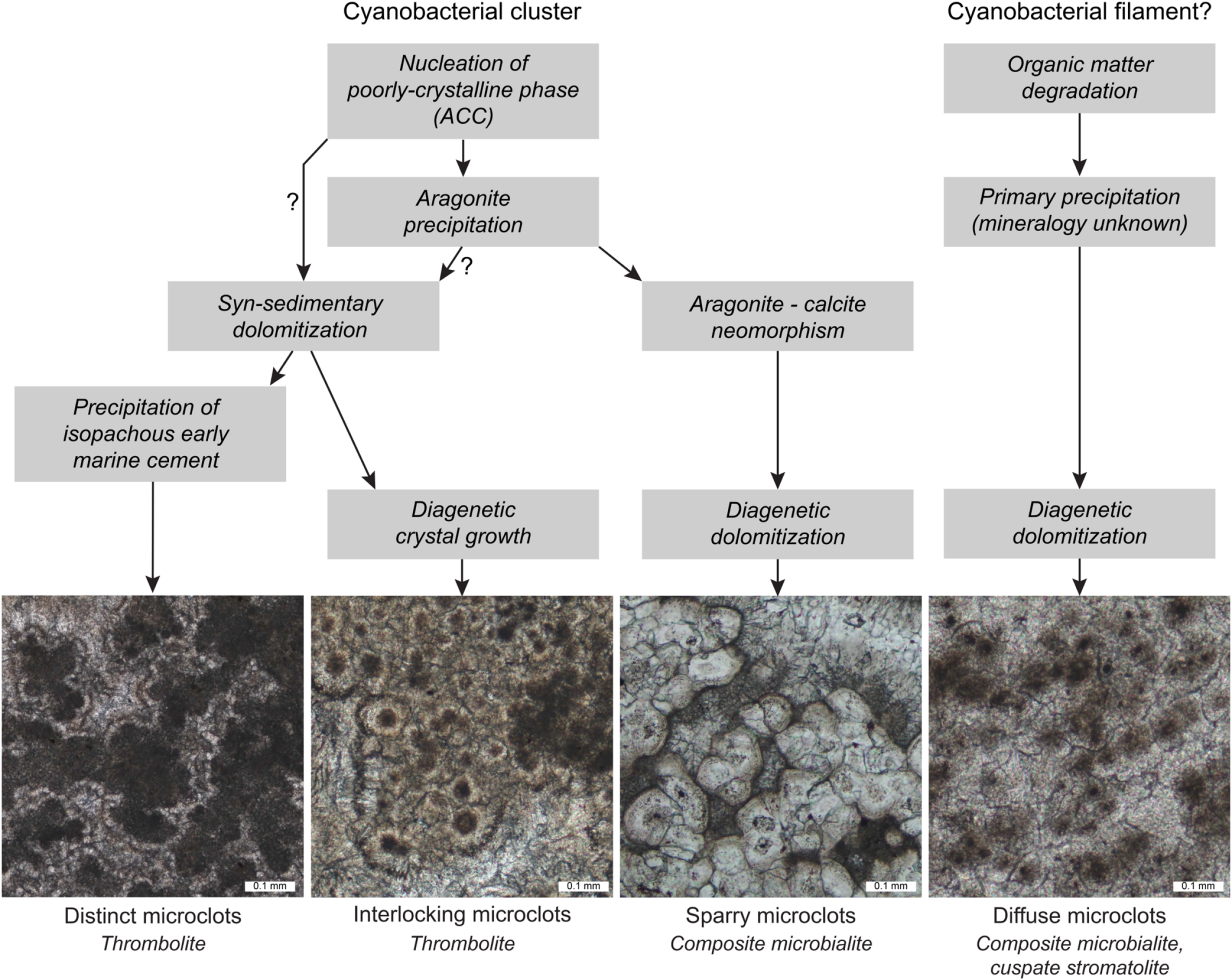
Schematic diagram of the inferred morphogenesis and paragenesis of endmember textures within the thrombolite, composite microbialite and cuspate microbialite. All petrographic photographs are at the same scale (field of view: 0.6 mm).

A key distinction to modern seawater in the REE + Y distribution pattern within the thrombolite facies is it lacks a clear negative Ce/Ce* anomaly. In fact, the Ce/Ce* anomaly of thrombolite marine cements is positive on average and has a larger range than other Neoproterozoic successions (1.42 ± 0.24, *n* = 41, range = 1.22; Figure 9). In the cuspate microbialite facies, the REE + Y distribution pattern is flatter, with a slight enrichment in the middle rare earth elements (MREE, i.e., Sm–Dy; Figure 8). The Ce/Ce* anomaly of the cuspate facies marine cements is 1.07 ± 0.19 (*n* = 49; Figure 10).

The cuspate microbialite facies displays a positive Eu anomaly (1.51 ± 0.37), while a smaller but still statistically significant positive Eu anomaly occurs in the thrombolite facies (1.15 ± 0.12; Figure S5).

## 5 Discussion

### 5.1 Cement Characterization

In the thrombolite, composite microbialite, and cuspate microbialite facies, microbialite cavities are coated by isopachous, inclusion‐rich, radial, fascicular cements which retain fine‐scale growth banding (Figure 3D,F,G). The precipitation of these cements predates the deposition of dolomicrite which we infer is detrital internal sediment, considering it is laminated and forms geopetal structures. The petrographic characteristics of the cements fit the well‐documented criteria for identification of marine cements (e.g., Bathurst 1972; Davies 1977). These marine cements form during the very earliest stages of diagenesis, when the system is sufficiently open with respect to marine fluids that the cements are precipitated in equilibrium with the prevailing seawater. In the Callison Lake Formation specifically, the presence of internal sediments within the marine‐cemented cavities implies a system that is so open to marine fluids that marine sediments can fall into the cavity system from the sediment–water interface. Therefore, the cements can be considered a record of syn‐sedimentary seawater. Based on modern tropical marine systems, it has been estimated that at least 100,000 pore volumes of fluid are required to completely fill pores in marine sediments (Scholle and Halley 1989). This suggests that marine cements like those in the Callison Lake Formation are precipitated under very active seawater advection through an open cavity system. Such a situation is most likely to occur in shelf margin settings in association with reef development (hence the strong association between marine cementation and reefal carbonates; Moore and Wade 2013).

The primary marine origin of the isopachous cements can also be demonstrated geochemically. Isopachous cements have the same REE patterns as marine carbonate sediments (internal sediment and ooids) (Figure S7), but lower concentrations of Th, ΣREE and Zr, which tend to be enriched in siliciclastic sediment (McLennan 1989; Figure S4). This demonstrates that the cements are primary marine precipitates and minimally affected by siliciclastic contamination. In fact, we note that all marine cement ablation spots for the Callison Lake Formation had Th, ΣREE and Zr values below the typical cut‐offs for marine cement analysis (Th < 0.1 ppm, ΣREE < 4 ppm, and Zr < 4 ppm; Stacey et al. 2023 and references within). Furthermore, in both facies, all Y/Ho values from marine cements are greater than 27, the Y/Ho value of PAAS.

Length‐slow optic character of marine cements has been considered indicative of precipitation as syn‐sedimentary dolomite (Hood and Wallace 2012, 2018). The Callison Lake Formation marine cements are dominated by thick, length‐slow dolomite cements with fine‐scale growth banding, similar to the Cryogenian Balcanoona Formation reef complexes of the Adelaide Superbasin (Hood and Wallace 2012). Therefore, the Callison Lake Formation can be considered another candidate for proposed syn‐sedimentary dolomite precipitation in the Neoproterozoic (Hood and Wallace 2018). We note that in the composite microbialite facies only, the earliest cements sometimes include a thin layer of length‐fast fibrous dolomite. This optic character is consistent with original precipitation as high magnesium calcite (Shuster et al. 2018).

Although the rim of cement coating the distinct microclots is too thin to preserve clear growth zonation, we suggest it is also an early stage of marine cement precipitation given its isopachous morphology. By contrast, the porosity‐occluding dolomite spar is typical of burial diagenesis, given that the crystals have rhombic habits and a strong red‐yellow luminescence response (Figure 3G).

### 5.2 Diagenetic Alteration

Microbialite microstructure is strongly affected by both organic matter degradation and diagenetic processes (e.g., Turner et al. 2000; Harwood and Sumner 2012). Therefore, much of the observed diversity in Callison Lake Formation microbialite texture may be derived from secondary alteration, rather than primary diversity. An example of alteration adding textural diversity is the interlocking microclots of the thrombolite facies. Within the thrombolite framework, there is a gradational relationship between distinct microclots and an increasingly cement‐rich texture, with interlocking microclot texture as the preservational endmember. Although these cements are radial, similar to the thin rim of marine cement surrounding the distinct microclots, we consider them to represent a secondary texture due to their inconsistent degree of cementation, variable sizes, subhedral shapes, and interfering rims. A secondary origin for the texture can also be inferred from the isolated nodules of interlocking microclot texture, which represent diagenetic crystallization fronts within the dominantly microcrystalline, organic‐rich framework (Figure 3E).

The sparry microclots in the composite microbialite show some similarities with the thrombolite interlocking microclots, namely their mutually‐interfering polygonal morphology with dark microcrystalline centres. However, there are some notable differences. The sparry microclots are comprised of larger crystals of spar with uniform extinction, and they have an open texture with small cavities between components. These features are similar in scale and polygonal geometry to “spherulites” within modern microbialites (figure 14 of Arp et al. 2012; figure 3d of Bischoff et al. 2020) and Jurassic thrombolites (figure 11F of Bosence and Gallois 2022), which are thought to form through aragonite growth in supersaturated, nucleation‐limited conditions (Arp et al. 2012). However, in the Callison Lake Formation, sparry microclots have uniform extinction rather than radial extinction “crosses”, therefore we suggest an alternative formation mechanism: non‐syntaxial replacement of the original carbonate fabric (Figure 10). This may have been a transition from aragonite to calcite, which results in characteristic overprinting of the original mineral fabric by calcite spar (Land 1967; Tucker 1984; Singh 1987; Zempolich and Baker 1993; Hood and Wallace 2018). The morphology of the sparry microclots also resembles guttulatic calcite texture described by Scheller et al. (2022), defined as having pseudo‐hexagonal or spherical cores with concentric ellipsoidal overgrowths. The texture was suggested to be characteristic of pseudomorphic replacement following ikaite dehydration (Scheller et al. 2022), however the intermediate phase may have also been one of the many other unstable carbonate minerals that have been recorded in modern settings (e.g., monohydrocalcite: Li et al. 2012, hydromagnesite and dypingite: Power et al. 2007; vaterite: Giralt et al. 2001).

In the stromatolitic parts of the composite and cuspate microbialites, microclots are small and diffuse (*sensu* Harwood and Sumner 2012) with indistinct boundaries. Diffuse microclots are smaller than the other microclots documented in our study (Figure 10), and this significant difference suggests these textures represent separate microbialite‐forming communities or processes, rather than preservational variants of the same original texture. However, given the consistently diffuse preservation of these stromatolite‐forming microclots, an origin for these structures cannot be determined from our study alone. We note that the Callison Lake diffuse microclots are the same in texture and size as the diffuse microclots of the Beck Spring Dolomite (Harwood and Sumner 2012, note figures 6C and 7C) and the “grumeaux” of the Little Dal Group (Turner et al. 2000). We therefore tentatively suggest that these microclots may have formed through the process suggested in these other case studies: organic degradation of filamentous, stromatolite‐forming cyanobacteria prior to lithification.

In the sinuous microbialite facies, the most notable alteration product is apatite, whose morphology resembles that of the microbialite structure in which it occurs, that is, either as polylobate protrusions like the microbialite framework, or as tabular layers like the internal sediment. Apatite formation must have occurred during early marine diagenesis; after microbialite growth had ceased, but before the internal sediment was brecciated (tabular apatite) and the cavities were completely filled by internal sediment and late‐stage cements (polylobate apatite).

The conversion of carbonate to apatite is likely to be induced by high fluid phosphate concentrations. There is evidence for high seawater phosphate in the Tonian, as determined by the phosphorus content of fine‐grained shales and carbonates (Planavsky et al. 2023; Roest‐Ellis et al. 2023) and the appearance of apatitic scale microfossils at this time (Riedman et al. 2021). However, given that microbially‐associated apatite is restricted to one approximately 5 m‐thick horizon of the sinuous microbialite facies, local processes must have played an important role. Pore water phosphate is increased by the respiration of sedimentary organic carbon (Guilbaud 2025 and references within). As apatite is restricted to the outer edge of the microbialite, we infer that the apatite formed authigenically as a result of respiration of organic carbon in internal sediment, adjacent dolosiltite, or even within the microbialite itself. More speculatively, we suggest that the restricted occurrence of apatite in the Callison Lake Formation may be explained by the metabolism of the specific microbial community that formed the sinuous microbialite. Microbial sulfate reduction is linked to substantial P enrichment of pore water, in part due to the release of Fe (oxyhydr)oxide‐bound P during pyrite formation (Slomp et al. 2002). This redox pathway may have been responsible for the apatization of the sinuous microbialite, perhaps superimposed upon high background concentrations of seawater phosphate.

### 5.3 Origin of Thrombolite Texture

In contrast to other, variably altered microbialite textures, the distinct microclots in the thrombolite facies are well‐preserved with a consistent size, shape, and composition, as well as distinct boundaries with other components. This suggests they are more similar to the original microbialite than the other endmember textures, and their original formation mechanism can be considered.

Thrombolite textures in some modern microbialites have been suggested to form through accumulation of detrital grains (Planavsky and Ginsburg 2009; Airo 2010; Chacón‐Baca et al. 2024) or disruption of an existing fabric by eukaryotes or dissolution‐reprecipitation cycles (Planavsky and Ginsburg 2009; Bernhard et al. 2013, 2023; Chacón‐Baca et al. 2024). Neither of these mechanisms is consistent with the texture of the Callison Lake Formation thrombolites, as microclots form an interconnected, three‐dimensional network without sharp boundaries and with a consistent size and shape. Furthermore, the consistency of the distinct microclot fabric and texture suggests that the microclots are unlikely to have formed through alteration of a more coarsely crystalline fabric. Micritization processes such as endolithic microboring generally preserve partially micritized components and destructive micrite envelopes (Chacón‐Baca et al. 2024; Garuglieri et al. 2024), which are not seen in the Callison Lake thrombolite microclots.

Instead, considering the consistent composition of the microclot fabric, we favour a formation mechanism involving direct, metabolism‐induced carbonate precipitation within an organic framework, as seen in numerous modern examples (Moore and Burne 1994; Arp et al. 2003; Airo 2010; Burne et al. 2014; Pace et al. 2016). Specifically, we suggest that Callison Lake thrombolite microclots formed by microcrystalline carbonate precipitation replacing a cluster of cyanobacteria within extracellular organic matter (EOM; Figure 10), through a similar mechanism to modern microbialites from Great Salt Lake (Pace et al. 2016). In these modern microbialites, cementation begins with the nucleation of poorly crystalline Mg‐Si phases within EOM, initiated by a rise in pH through photosynthesis. Subsequently, small aragonite crystals precipitate, first within coccoid cyanobacterial cells, then around cell walls and Mg‐Si crystals, replacing the organic framework (Pace et al. 2016). In our ancient samples, we see no evidence of Mg‐Si phases such as clay minerals, but a similar mechanism of fine‐scale cementation at a consistent nucleation rate is compelling, as it would account for the homogenously microcrystalline fabric and the inclusion of organic matter. The precursor phase may have been a poorly crystalline carbonate phase such as amorphous calcium carbonate (ACC), which would have transformed to a more stable mineralogical phase. It has been shown that high magnesium ACC can directly transform to disordered dolomite (Rodriguez‐Blanco et al. 2015; Liu et al. 2024). Alternatively, ACC can transform to monohydrocalcite or aragonite (Methley et al. 2025). Since mimetic replacement of microcrystalline textures can occur through direct replacement of aragonite by dolomite (Zempolich and Baker 1993), an intermediate step of aragonite formation (Figure 10) is not ruled out by textural relationships.

Distinct microclot texture has been recognised in the Beck Spring Dolomite (figure 9 of Harwood and Sumner 2011; figure 7 of Harwood and Sumner 2012; figure 10B/D of Shuster et al. 2018) and there is evidence for distinct, cement‐rimmed microclots within the Devede Formation (figure 12 of Hood et al. 2015). The same microclots seem to be absent from older thrombolitic carbonates, and this may be due to the replacement dolomitization of some pre‐Tonian microbialites, which obscures the original texture in petrographic thin section (Kah and Grotzinger 1992; Tang et al. 2013). In fact, compared to earlier Precambrian examples, microbialites in the Callison Lake Formation, Beck Spring Dolomite and Devede Formation are exceptionally well‐preserved. The mimetic dolomitization of these thrombolites, like the syn‐sedimentary dolomitization of the marine cements in the same facies (Hood et al. 2015; Shuster et al. 2018; Stacey et al. 2023; this study), may be attributable to the unique, Mg‐rich chemistry of late Tonian seawater.

### 5.4 Depositional Context

In Phanerozoic metazoan‐dominated reefs, isopachous fibrous marine cements occupy a large amount of framework space and are considered characteristic of reefal facies (James and Choquette 1983; Wood 1999). Marine cements significantly contribute to growth framework rigidity (James and Choquette 1983), which is a defining characteristic of reefs (e.g., Lowenstam 1950) and necessary for a biological community to construct significant relief above the surrounding seafloor. For example, in the Devonian Canning Basin, radiaxial fibrous marine cement commonly makes up 20%–50% of reefal boundstones by volume (Kerans et al. 1986), and similarly, rigidity in the late Permian Capitan reef is attributed to early cementation of fibrous calcite, which accounts for up to 70% of reef volume locally (Wood et al. 1996). This Phanerozoic‐style marine cementation within a porous framework is also a defining characteristic of Neoproterozoic reefs (Wood 1999; Wallace et al. 2015). Other substantial Precambrian reef frameworks are not porous or cemented, but reef‐building platforms are identified by their thick, laterally extensive successions of microbialite (Cecile and Campbell 1978; Grotzinger 1986; Turner et al. 1997).

In the Callison Lake Formation, there are ubiquitous, thick marine cements within microbialite growth frameworks. The prevalence of cements implies that the Callison Lake Formation microbialites were open to seawater flushing through for an extended period, and that these facies were equivalent to Phanerozoic reef boundstones in terms of their structural rigidity. In addition, in the most complete sections of the Ramp member (CAL‐1, Figure 2), there are thick (> 50 m) successions of microbialite, following a general trend of complex unlaminated microbialite frameworks grading upwards into large‐scale stromatolitic frameworks. This is a similar facies succession to that found in the Cryogenian Balcanoona Reef complexes of South Australia (Wallace et al. 2015). The overall similarity in vertical succession with the Cryogenian Balcanoona reefs, the very large volume of growth framework facies and the strongly marine‐cemented nature of the Ramp member are all suggestive of a relief‐building reef system.

Within the Mount Harper section, the sinuous microbialite is laterally continuous with fenestral dolosiltstone, as well as clasts of breccia containing polygonal cracks. Both fenestrae and cracks are likely to have formed through space creation by dehydration, which may be related to either periodic exposure or the transformation of a hydrated mineral under subaqueous conditions. Given the presence of cross‐stratified dolosiltstone and breccia in the same facies, we suggest the sinuous microbialite formed in a shallow peritidal environment in the uppermost part of the reef, where it was affected by both periods of emergence and relatively high water energy. The composite microbialites were likely deposited in a similar environment, given their association with oncoids which have asymmetric dissolution textures that may be related to exposure or to meteoric dissolution. In contrast, in the same section, the thrombolite facies is overlain by cross‐bedded grainstone and domal stromatolites. These relationships imply a slightly deeper environment than the sinuous microbialite facies, and we suggest the thrombolites formed in a shallow environment affected by wave action, that is, close to fair‐weather wave base.

The cuspate microbialites in the Mount Gibben section are associated with dolomudstone interbedded with intraclastic breccia (Figure 2). In this setting, dolomudstone likely represents offshore deposition free from wave action, while intraclastic breccias accumulated via gravity flows, which are characteristic of reef slope settings (Mcllreath and James 1978). Therefore, we suggest the cuspate microbialites in the lower part of the Ramp member grew as deeper water reefal frameworks, likely well below fair‐weather wave base. The deeper depositional setting of the cuspate microbialites compared to the thrombolites aligns with the broader stratigraphic context, which suggests regional deepening to the east (Strauss et al. 2015). Cuspate microbialites have also been found in deep‐water settings in other Precambrian carbonate successions (e.g., Sami and James 1993; Bartley et al. 2015; O'Connell et al. 2022).

### 5.5 Redox Setting

Petrographic and geochemical observations provide strong evidence that the isopachous cements targeted for laser ablation are primary, syn‐sedimentary marine cements which formed in equilibrium with the open ocean (Section 5.1). Marine cements have been shown to be excellent archives of redox‐dependent trace element and rare earth element (REE) geochemistry (Nothdurft et al. 2004), therefore fine‐scale, component‐specific analysis can provide insight into the redox geochemistry of ancient seawater. This type of analysis has previously been used to reconstruct the redox geochemistry of seawater associated with other Neoproterozoic formations, including the ca. 750 Ma Beck Spring Dolomite (California, USA; Shuster et al. 2018), the ca. 760 Ma Devede Formation (Namibia; Stacey et al. 2023), the ca. 650 Ma Balcanoona Formation (Australia; Hood and Wallace 2014, 2015), and the ca. 635 Ma Nuccaleena Formation (Australia; Lamothe et al. 2024).

In the Callison Lake Formation, cement geochemistry indicates that the thrombolite and cuspate microbialite formed within predominantly anoxic seawater. The redox‐sensitive elements U and V are soluble in their oxidized forms, but under anoxic conditions, they are reduced to insoluble states or form complexes with reduced species, resulting in their removal from seawater (Anderson et al. 1989; Emerson and Huested 1991; Klinkhammer and Palmer 1991; Algeo and Maynard 2008). The extent of anoxia in a basin may therefore be inferred by the enrichment of these elements in sediments, and their corresponding depletion in seawater (e.g., Partin et al. 2013). Marine cements of the thrombolite and cuspate microbialite facies, which record seawater values, have very low concentrations of U and V (Figure 7), particularly when compared to modern carbonates that have U concentrations on the order of several ppm (Gvirtzman et al. 1973; Romaniello et al. 2013) and V concentrations on the order of 10s of ppm (Hastings et al. 1996). This indicates strong anoxia in both shallow (thrombolite facies) and comparatively deep (cuspate microbialite facies) settings.

Positive Eu anomalies occur in both facies, which also adds to the evidence for anoxic seawater conditions. Positive Eu anomalies are considered a proxy for hydrothermal input to seawater, as Eu(II) is mobile in acidic, high temperature conditions and is therefore enriched in hydrothermal fluids relative to the other REEs (Elderfield 1988; Bau 1991). This anomaly is only retained throughout an ocean basin if seawater is predominantly anoxic, because in oxygenated conditions such as the modern ocean, excess Eu(II) is oxidized to Eu(III) and scavenged by Mn‐Fe (oxyhydr)oxides (Bau 1991).

As well as general anoxia, marine cement trace element geochemistry provides evidence for specifically euxinic (anoxic, H_2_S‐bearing) seawater conditions. In the Callison Lake Formation, there are strong correlations between chalcophile elements in both the thrombolite facies (Co–Cu–Zn–Pb) and the cuspate microbialite facies (Cd–Zn and Zn–Pb), indicating a common mechanism of depletion. Concentrations of these chalcophile elements are significantly lower (at a confidence interval of 0.05) than in the ferruginous deep‐water facies of the Cryogenian Balcanoona Formation (Hood and Wallace 2015) and the oxic shallow‐water facies of the Ediacaran Nuccaleena Formation (Lamothe et al. 2024; Figure 7, Table S2). Furthermore, chalcophile element concentrations in the cuspate microbialite facies are generally less than or statistically indistinguishable from marine cements of the Beck Spring Dolomite, which has been interpreted to record a dominantly euxinic environment (Shuster et al. 2018; Figure 7, Table S2). Overall, these results suggest that the chalcophile elements were drawn down locally from seawater by incorporation into sulfides, as expected under euxinic conditions (van der Weijden 1993; Cooper and Morse 1998; Tribovillard et al. 2006), leaving seawater depleted in these elements. Furthermore, the Fe concentrations in the cuspate microbialite facies are significantly lower than the ferruginous Balcanoona Fm. This fits the interpretation of euxinic conditions, in which Fe would be removed from the water column through reaction with freely available dissolved sulfide. Despite evidence for widespread ferruginous deep water during the Tonian period both in the Yukon (Sperling et al. 2013; Gibson et al. 2020) and globally (Guilbaud et al. 2015), the depletion of chalcophile elements in the Callison Lake Formation is consistent with other studies documenting euxinia was also present in late Tonian oceans (Dahl et al. 2011; Shuster et al. 2018; Stacey et al. 2023).

Microbialites at different interpreted depths have marine cements with different REE + Y distribution patterns (Figure 8), which indicates this basin was redox stratified. In the shallow thrombolite facies, marine cements preserve a positive Ce/Ce* anomaly, a REE + Y distribution pattern with light rare earth element (LREE; La–Nd) depletion, and a positive Y/Ho anomaly greater than 36 (Figures 8, 9, and S5). By contrast, the comparatively deep cuspate microbialite facies of the Callison Lake Formation does not have a significant Ce/Ce* anomaly and features a flattened, MREE‐enriched distribution pattern similar to that of the Beck Spring Dolomite (Figures 8 and 9). Both distribution patterns are different from the typical seawater REE + Y profile of a fully oxygenated ocean, in which Ce is preferentially sequestered from the water column by Mn‐Fe (oxyhydr)oxide adsorption (Bau 1999; Bau and Koschinsky 2009), resulting in a distinct negative Ce/Ce* anomaly (mean of 0.36; Wallace et al. 2017).

The positive Ce/Ce* anomaly of the thrombolite facies likely reflects the regeneration of Mn‐Fe (oxyhydr)oxide‐bound Ce. If the thrombolite facies were well below a chemocline, we would expect full dissolution of Mn‐Fe (oxyhydr)oxides, which are enriched in Ce relative to other REEs, depleted in HREEs, and depleted in Y/Ho (Bau and Koschinsky 2009). However, this is not consistent with the REE + Y distribution pattern of the marine cements in this facies, which is LREE‐depleted and has a positive Y/Ho within the range of modern seawater. Therefore, we suggest the thrombolite facies was deposited within the chemocline itself, where Mn‐Fe (oxyhydr)oxides were not fully dissolved and underwent cycles of regeneration and reprecipitation. Preferential release of Ce compared to other REEs is observed in the upper sediment column of Buzzard Bay (Massachusetts, USA), associated with dissolution‐reprecipitation cycles of Mn‐Fe (oxyhydr)oxides within pore water (Elderfield and Sholkovitz 1987). A similar mechanism may be applicable to the Callison Lake Formation, with a subtly fluctuating depth of the chemocline responsible for cycling Mn‐Fe (oxyhydr)oxides. This interpretation of precipitation within a chemocline also explains the Fe enrichment in this facies, which would result from the dissolution of oxidized Fe species.

In modern oceans, the minimum oxygen levels required to oxidize Mn (II) are on the order of several μM (Tebo 1991; Clement et al. 2009), and similarly, Fe (II) is oxidized and sequestered from seawater in oxic to suboxic conditions (Landing and Bruland 1987; Hopkinson and Barbeau 2007). The existence of a Mn‐Fe (oxyhydr)oxide shuttle therefore implies the upper mixed layer was at least suboxic (following the definitions of Tyson and Pearson 1991). This (sub)oxic layer must have been very shallow, likely confined to the uppermost few metres of the water column, as it lay above the shallow marine setting of the thrombolite facies.

By contrast, marine cements in the cuspate microbialite have a flattened REE + Y distribution and low Y/Ho values, between that of modern seawater and PAAS. This pattern indicates complete remineralization of REE‐bearing phases such as Mn‐Fe (oxyhydr)oxides and organic carbon, in a process analogous to the generation of flattened pore water REE + Y profiles below the oxic layer in modern sediments (Elderfield and Sholkovitz 1987; Sholkovitz et al. 1992; Haley et al. 2004; Himmler et al. 2013; Smrzka et al. 2019). However, in an anoxic stratified ocean, remineralization would occur within the water column, generating a flattened seawater REE + Y profile with low Y/Ho below the chemocline. Similar profiles are observed in modern anoxic basins such as the Black Sea (Schijf et al. 1991) and the Tyro sub‐basin (Bau et al. 1997). We emphasize here that in an anoxic stratified ocean, a flattened REE + Y distribution with Y/Ho < 36 is not necessarily a diagnostic criterion of siliciclastic contamination, freshwater influence or diagenetic alteration.

On the basis of these geochemical characteristics and microbialite facies analysis, cuspate microbialites were likely deposited well below the chemocline, within dominantly euxinic water. Their cement REE + Y distribution pattern is also typical of Tonian seawater in other localities, such as the Devede Formation and the Beck Spring Dolomite (Shuster et al. 2018; Stacey et al. 2023). Our interpretation of a redox‐stratified anoxic ocean is comparable to previous work on the Cryogenian Balcanoona Formation and the Ediacaran Nuccaleena Formation, whose marine cement REE + Y distribution patterns vary with paleo‐depth (Hood and Wallace 2015; Lamothe et al. 2024).

Despite the evidence for dominantly euxinic conditions in shallow settings, the wide range of Ce/Ce* values for both the cuspate microbialite and thrombolite primary marine cements likely reflects a degree of redox variability. A range of Ce/Ce* implicates locally fluctuating oxygen levels on the timescale of cement growth zonation (Stacey et al. 2023), which in this case may reflect small fluctuations in the position of the very shallow chemocline (e.g., due to variability in upwelling or mixing of the surface ocean). This effect is particularly pronounced for the cements in the thrombolite facies, which have a larger Ce/Ce* range than any other Neoproterozoic primary marine cements (1.22; Figure 9). The range in Ce/Ce* values is consistent with the inferred proximity of the thrombolite facies to the chemocline. Alternatively, periods of partial oxygenation within the thrombolite facies could be partly attributed to oxygenic photosynthesis. While both microbialite facies likely had oxygenic photosynthesizers, seawater advection within the open, porous thrombolite framework could have facilitated local oxygenation much more readily than in the lower porosity stromatolite facies.

Our results bear on the Neoproterozoic Oxygenation Event (NOE), which canonically involves a step‐wise increase in oxygen, variably inferred to occur during the late Neoproterozoic (e.g., Canfield et al. 2007) or begin circa 800 Ma (Cole et al. 2016; Wang et al. 2022). Others have argued that the Neoproterozoic oxygenation was non‐linear, characterized by short‐term oxygenation events and spatial heterogeneity (Reinhard et al. 2016; Tostevin and Mills 2020; Krause et al. 2022), with full ocean oxygenation delayed until the middle Paleozoic (Wallace et al. 2017; Stockey et al. 2024). We demonstrate that the Callison Lake Formation was deposited in a marine basin where shallow marine environments were anoxic and euxinic, and (sub)oxic surface water was restricted to very shallow depths above a redoxcline. These redox conditions are reminiscent of multiple studies of Mesoproterozoic basins where dominantly anoxic basinal conditions are interpreted to be overlain by oxygenated surface water (Gilleaudeau and Kah 2015; Cox et al. 2016; Doyle et al. 2018). By contrast, early to middle Tonian shales in the Yukon block are interpreted to have ferruginous water below a chemocline, but with episodic oxygenation of deeper waters (Sperling et al. 2013; Thomson et al. 2015; Gibson et al. 2020; Maloney et al. 2024), at a time corresponding to an increase in global oxygenation ca. 800 Ma (Cole et al. 2016; Wang et al. 2022; Webb et al. 2026). The prevalence of shallow marine anoxia and euxinia in the late Tonian (Shuster et al. 2018; Stacey et al. 2023; this study) suggests that any middle Tonian oxygenation was not permanent, but rather part of dynamic, protracted, and distinctly non‐unidirectional Neoproterozoic oxygenation.

## 6 Conclusions

Callison Lake Formation microbialites are diverse at both the mesoscale and microscale, and they are constructed by a diverse range of endmember textures. The balance of petrographic and geochemical evidence suggests that no single, universal process can account for the thrombolite (and other unlaminated microbialite) textures. Specifically, late‐stage processes such as diagenesis, recrystallization, and non‐syntaxial replacement can significantly contribute to the final texture in ancient microbialites.

Notwithstanding the role of variable diagenesis in the thrombolite textures, the rocks examined in this study are generally exceptionally well‐preserved compared to the extensively recrystallized pre‐Tonian examples of thrombolites (Kah and Grotzinger 1992; Tang et al. 2013). Like the late Tonian Beck Spring Dolomite (Harwood and Sumner 2012) and Devede Formation (Hood et al. 2015; Stacey et al. 2023), the Callison Lake Formation thrombolites form distinct microcrystalline clots with a framework texture and a consistent fabric, which are likely to be close to the original microbialite texture. All three formations also have length‐slow isopachous marine cements with fine‐scale growth banding (Hood et al. 2015; Shuster et al. 2018; Stacey et al. 2023; this study), which are features interpreted to characterize syn‐sedimentary dolomite precipitation (Hood and Wallace 2018). We therefore posit that the exceptional preservation in these microbialites can be attributed to early, mimetic dolomitization indicating a unique preservational window linked to Mg‐rich late Tonian seawater chemistry. Further work on ancient microbialite case studies is needed to test this hypothesis and constrain the overall temporal trends in the unlaminated microbialite record.

We have also demonstrated that the Callison Lake Formation microbialites grew in a basin where (sub)oxic conditions were restricted to very shallow depths, likely only the upper few metres of the water column. The microbialites themselves formed in dominantly euxinic seawater, either within (thrombolite) or below (cuspate microbialite) the water column redoxcline. While other work has demonstrated an increase in global oxygen levels ca. 800 Ma (Cole et al. 2016; Wang et al. 2022; Webb et al. 2026), even shallow marine environments in the ca. 745 Ma Callison Lake Formation are dominantly anoxic. This study therefore adds to the evidence for non‐unidirectional or spatially variable oxygenation during the Neoproterozoic.

## Funding

This work was supported by the Natural Sciences and Engineering Research Council of Canada, RGPIN2023‐03514.

## Conflicts of Interest

The authors declare no conflicts of interest.

## Supporting information


**Figure S1:** Schematic diagram of the terminology for describing microbialites at different scales, as per the definitions and recommendations of Grey and Awramik (2020).
**Figure S2:** Additional photographs of Callison Lake Formation lithologies.
**Figure S3:** Petrography and electron dispersive spectroscopy (EDS) of opaque phases in the Callison Lake Formation thrombolite distinct microclots.
**Figure S4:** Crossplot of thorium and total REE concentrations of marine cements.
**Figure S5:** Crossplot of Y/Ho and Eu/Eu* ratios of marine cements.
**Figure S6:** Correlation matrix for the trace element concentrations of marine cements.
**Figure S7:** Average rare earth element plus Yttrium (REE + Y) distribution patterns for analyzed components in Callison Lake Formation microbialites, normalized to post‐Archean average shale (McLennan 1989).
**Table S1:** Definitions for microbialite‐specific descriptive terms used in this study.
**Table S2:** Pairwise Welch's unequal‐variances t‐tests comparing primary marine cement concentrations from different Neoproterozoic formations.

**Table S3:** LA‐ICP‐MS data (in ppm) for marine cements of the Callison Lake Formation.

## Data Availability

Data supporting the findings of this study are available in the Supporting Information of this article.
