# Supplementary material for: Microbialite Diversity and Ocean Redox Geochemistry of the Late Tonian Callison Lake Formation
Source: Geobiology. 2026 Jun 8;24(3):e70054. doi: 10.1111/gbi.70054 (PMC13244416; doi:10.1111/gbi.70054)
Supplement: Supplementary file 1 — Figure S1: Schematic diagram of the terminology for describing microbialites at different scales, as per the definitions and recommendations of Grey and Awramik (2020). Figure S2: Additional photographs of Callison Lake Formation lithologies. Figure S3: Petrography and electron dispersive spectroscopy (EDS) of opaque phases in the Callison Lake Formation thrombolite distinct microclots. Figure S4: Crossplot of thorium and total REE concentrations of marine cements. Figure S5: Crossplot of Y/Ho and Eu/Eu* ratios of marine cements. Figure S6: Correlation matrix for the trace element concentrations of marine cements. Figure S7: Average rare earth element plus Yttrium (REE + Y) distribution patterns for analyzed components in Callison Lake Formation microbialites, normalized to post‐Archean average shale (McLennan 1989). Table S1: Definitions for microbialite‐specific descriptive terms used in this study. Table S2: Pairwise Welch's unequal‐variances t‐tests comparing primary marine cement concentrations from different Neoproterozoic formations. [file GBI-24-e70054-s001.docx]

Supplement to: Microbialite diversity and ocean redox geochemistry of the late Tonian Callison Lake Formation

Reference details for each citation are provided in the main manuscript.


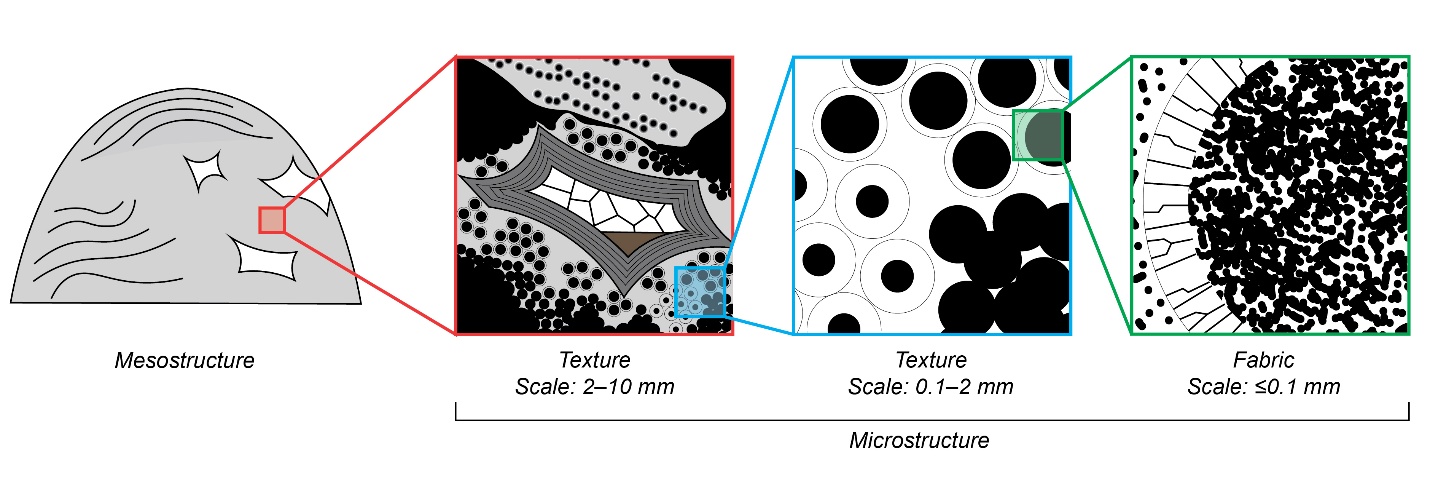


*Figure S1:* Schematic diagram of the terminology for describing microbialites at different scales, as per the definitions and recommendations of Grey and Awramik (2020).

| **Term** | **Definition (Grey and Awramik, 2020)** | **Adjustments and new definitions (this study)** |
| --- | --- | --- |
| Microbialite | Organosedimentary deposits that have accreted as a result of a benthic microbial community trapping and binding detrital sediment and/or forming the locus of mineral precipitation. |  |
| Stromatolite | A laminated organosedimentary structure produced by precipitation, or by sediment trapping and binding, as a result of the growth, behaviour, and metabolic activity of microorganisms, principally cyanobacteria. |  |
| Thrombolite | Microbialite composed of a clotted mesostructure. |  |
| Morphology | N/A | A general term used in this manuscript to encompass all distinguishing characteristics of microbialites, including mesostructure, texture, and fabric. |
| Facies | N/A | Where used in a microbialite-specific capacity, this refers to a category of microbialite defined by distinctive morphology (i.e. including mesostructure, microstructure, texture and fabric). |
| Mesostructure | Intermediate-scale features (between macrostructure and microstructure) that comprises the internal structure visible to the unaided eye. It is at this level that thrombolites, dendrolites, and leiolites show their distinction from stromatolites. | See Fig. S1. |
| Microstructure | A term restricted to those features best studied under the microscope and including texture, fabric and microfossils. | See Fig. S1. |
| Texture | The size, shape, and arrangement (packing and fabric) of the component elements of a sedimentary rock. | This term may be used at multiple microscopic scales. In this manuscript, we use texture to refer to arrangement of components on a cm-scale (e.g. partitioning of stromatolitic vs thrombolitic microbialite on the scale of a whole thin section) as well as a mm-scale (e.g. size, shape and arrangement of individual microclots). See Fig. S1. |
| Fabric | The orientation (or lack of it) of discrete particles, crystals and cement. … [Fabric is] a *microstructural* feature and a component of texture. | See Fig. S1. |
| Mesoclot | Millimetre- to centimetre-size spheroidal to polylobate masses composed of one to a variety of components (peloids, cement, grumeaux, calcimicrobes) within the groundmass of an unlaminated microbialite. |  |
| Microclot | Millimetre or smaller clot that is a constituent of a macroclot or is an isolated clot. | Grey and Awramik (2020) prefer the term miniclot. However, in our samples microclot is a more appropriate term to describe the individual constituents of clotted textures, given that they cannot be seen with the naked eye and are an exclusively microstructural feature. |

*Table S1:* Definitions for microbialite-specific descriptive terms used in this study.


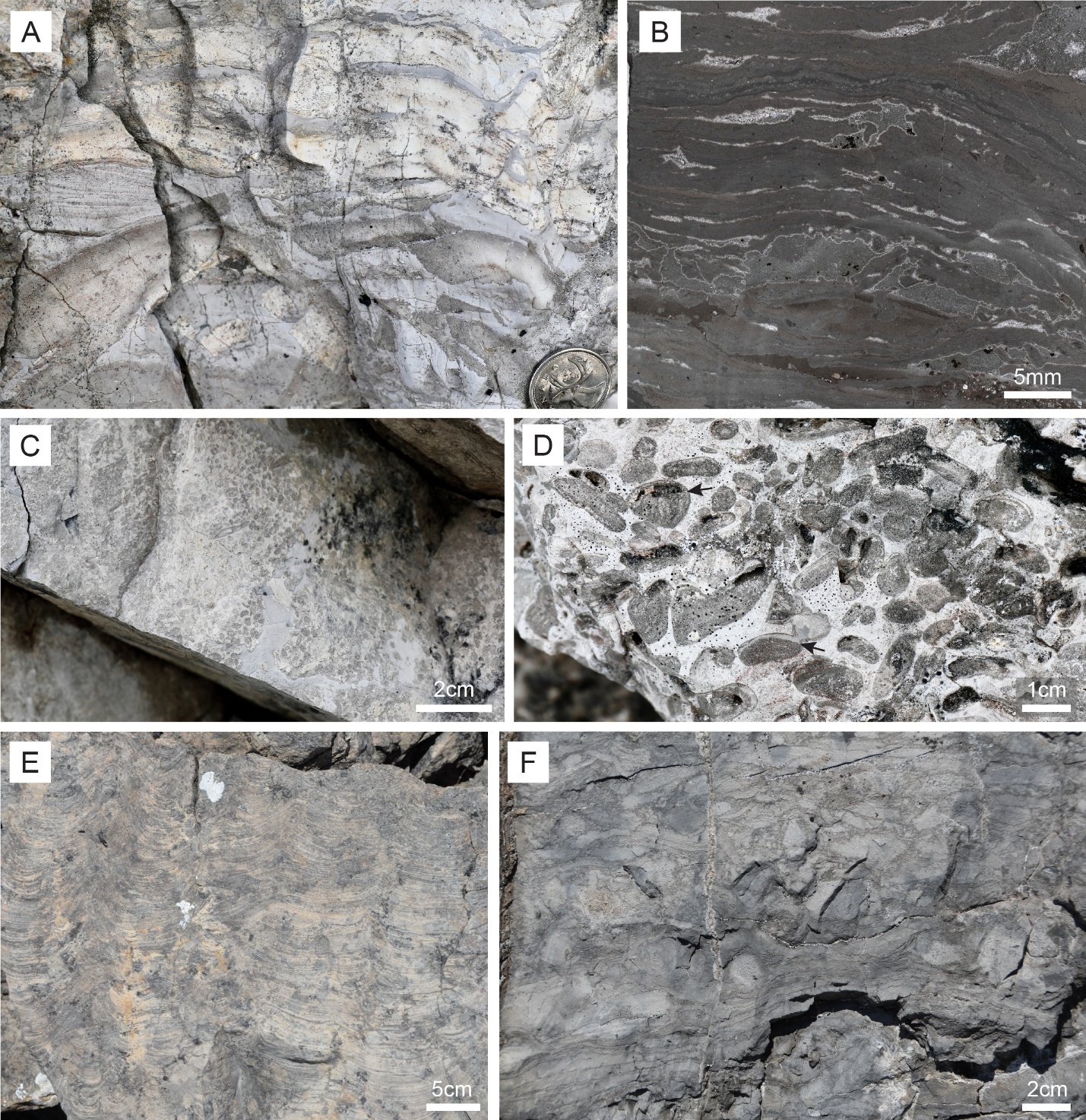


*Figure S2:* Additional photographs of Callison Lake Formation lithologies. (A) Outcrop photograph of cross-stratified dolosiltite with internal breccia associated with the fenestral microbialite facies. Canadian quarter for scale is 2.4 mm. (B) Plane-polarized light petrographic image of fenestrae within dolomudstone. (C) Outcrop photograph of peloidal rudstone associated with the thrombolite facies. (D) Outcrop photograph of oncoidal grainstone associated with the composite microbialite facies. Arrows denote asymmetric partial-dissolution textures. (E) Outcrop photograph of cuspate stromatolite, unannotated version of Fig. 6A. (F) Outcrop photograph of debris flow deposit (“debrite”) associated with the cuspate stromatolite facies.


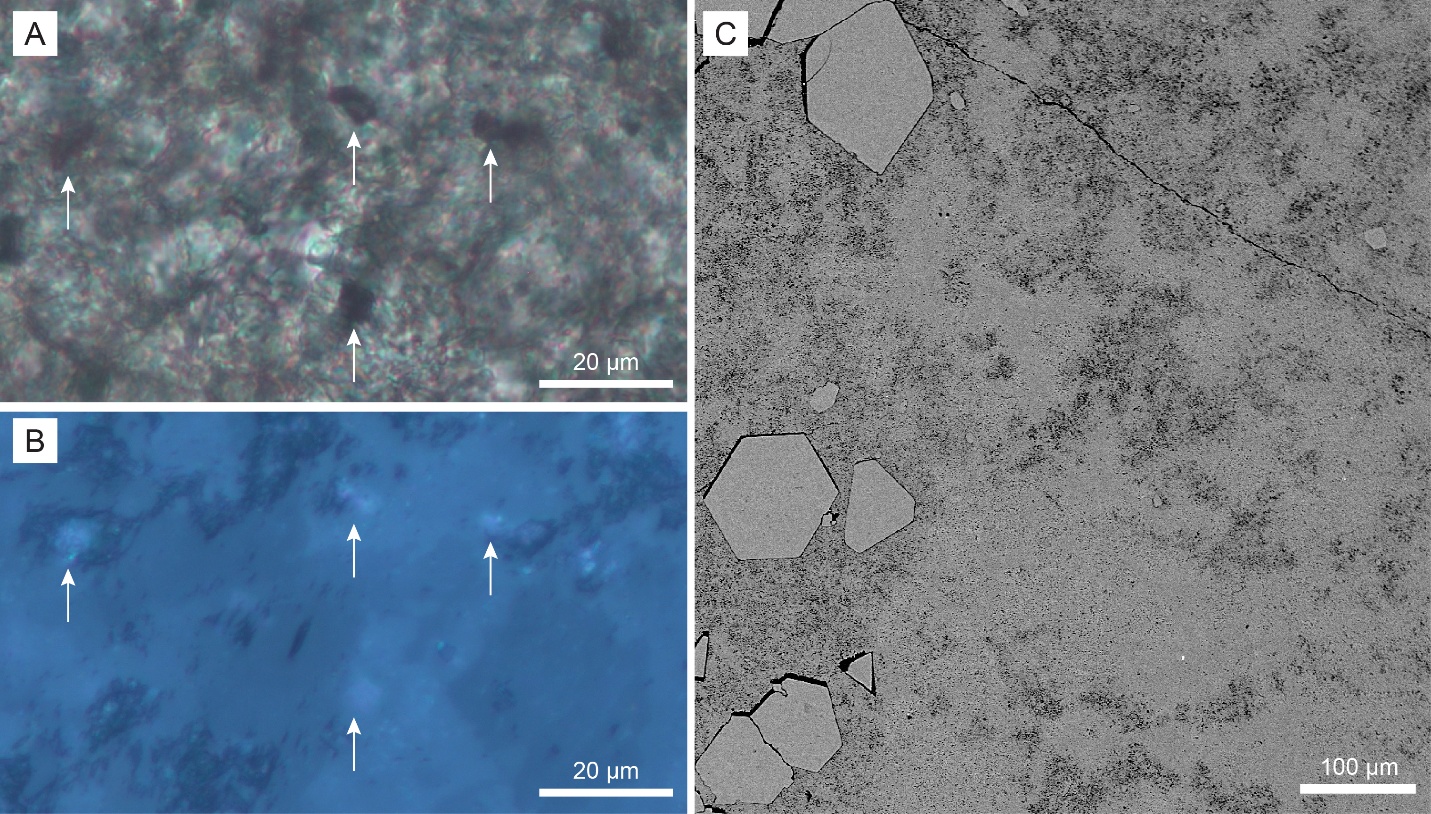


*Figure S3:* Petrography and electron dispersive spectroscopy (EDS) of opaque phases in the Callison Lake Formation thrombolite distinct microclots. (A) Plane polarized light image of the distinct microclot fabric. Arrows denote opaque phases described in the manuscript. (B) Same field of view as A, in reflected light with a white card. (C) EDS image from an area of thrombolite with a distinct microclot texture. The left-hand side of the image includes minor late-stage quartz crystals.

*
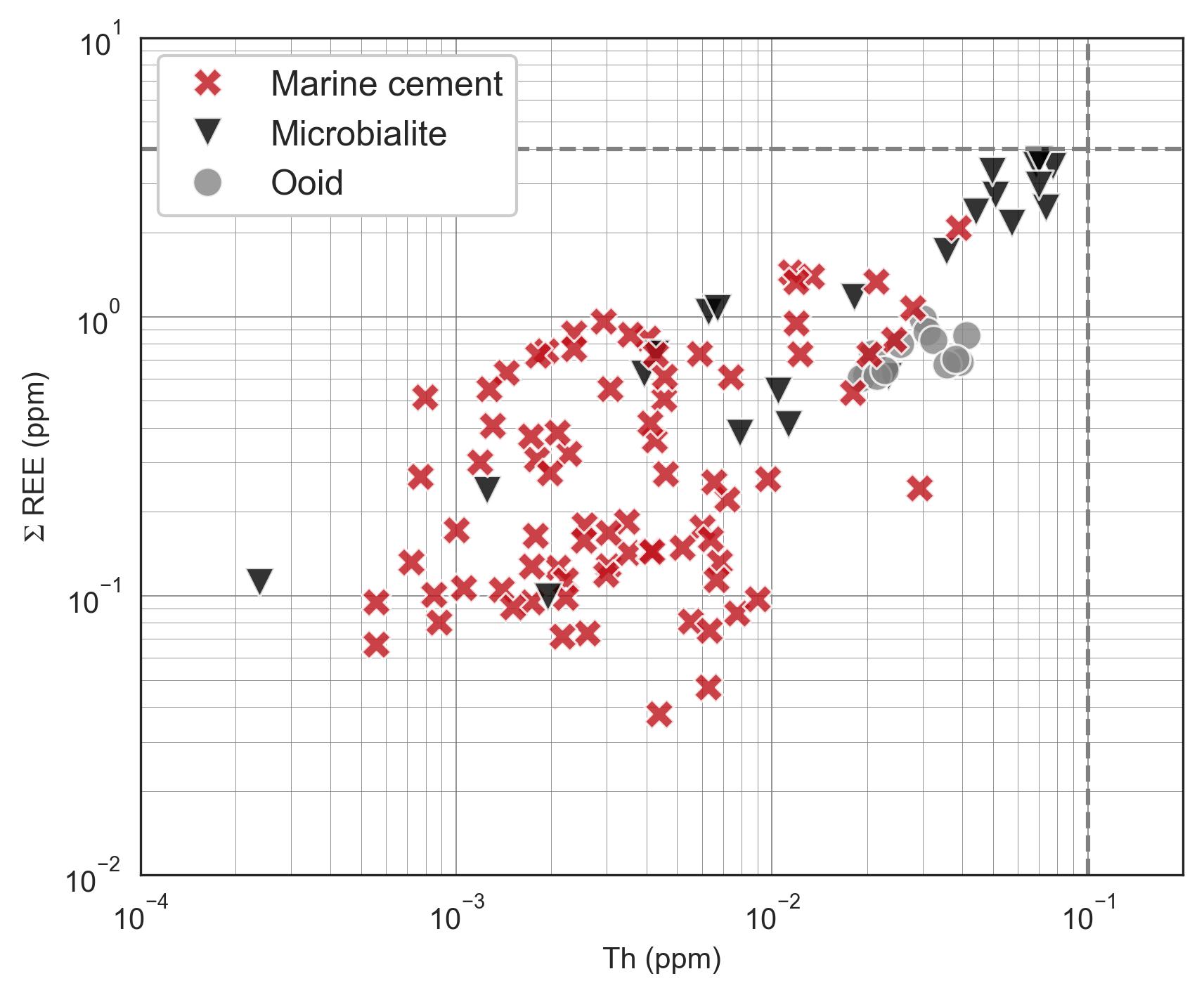
*

*Figure S4*: Crossplot of LA-ICP-MS data for thorium and total REE. Data is from marine cements, microbialites and ooids from the thrombolite and cuspate microbialites facies of the Callison Lake Formation. Dashed lines indicate typical lower cutoff values used to screen for detrital contamination (Th < 0.1 ppm, total REE < 4.0 ppm; Stacey *et al.* 2023 and references within).


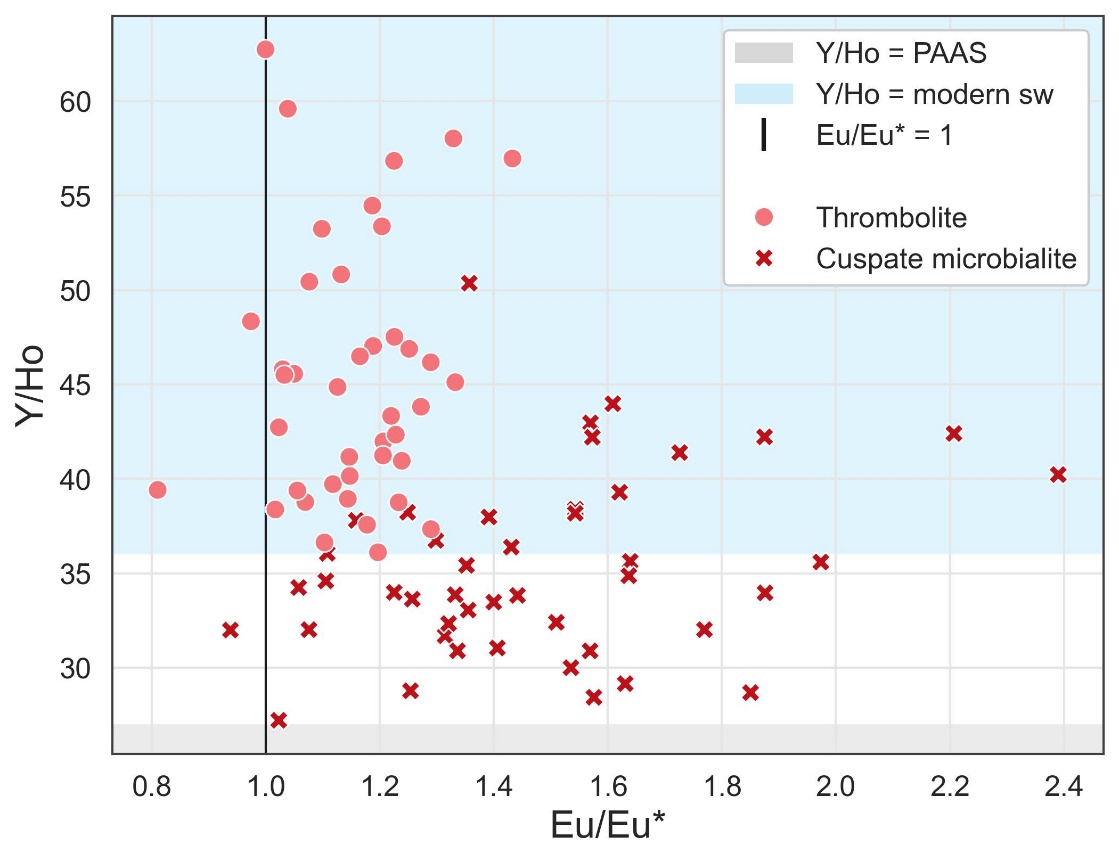


Fig. *S5*: Crossplot of LA-ICP-MS data for Y/Ho and Eu/Eu* ratios. Data is from marine cements from the thrombolite and cuspate microbialites facies of the Callison Lake Formation. Shaded regions represent the typical values of Y/Ho in Post-Archaean Average Shale (Y/Ho = 27; McLennan, 1989) and modern seawater (Y/Ho > 36; Bau et al. 1997; Censi et al. 2007).


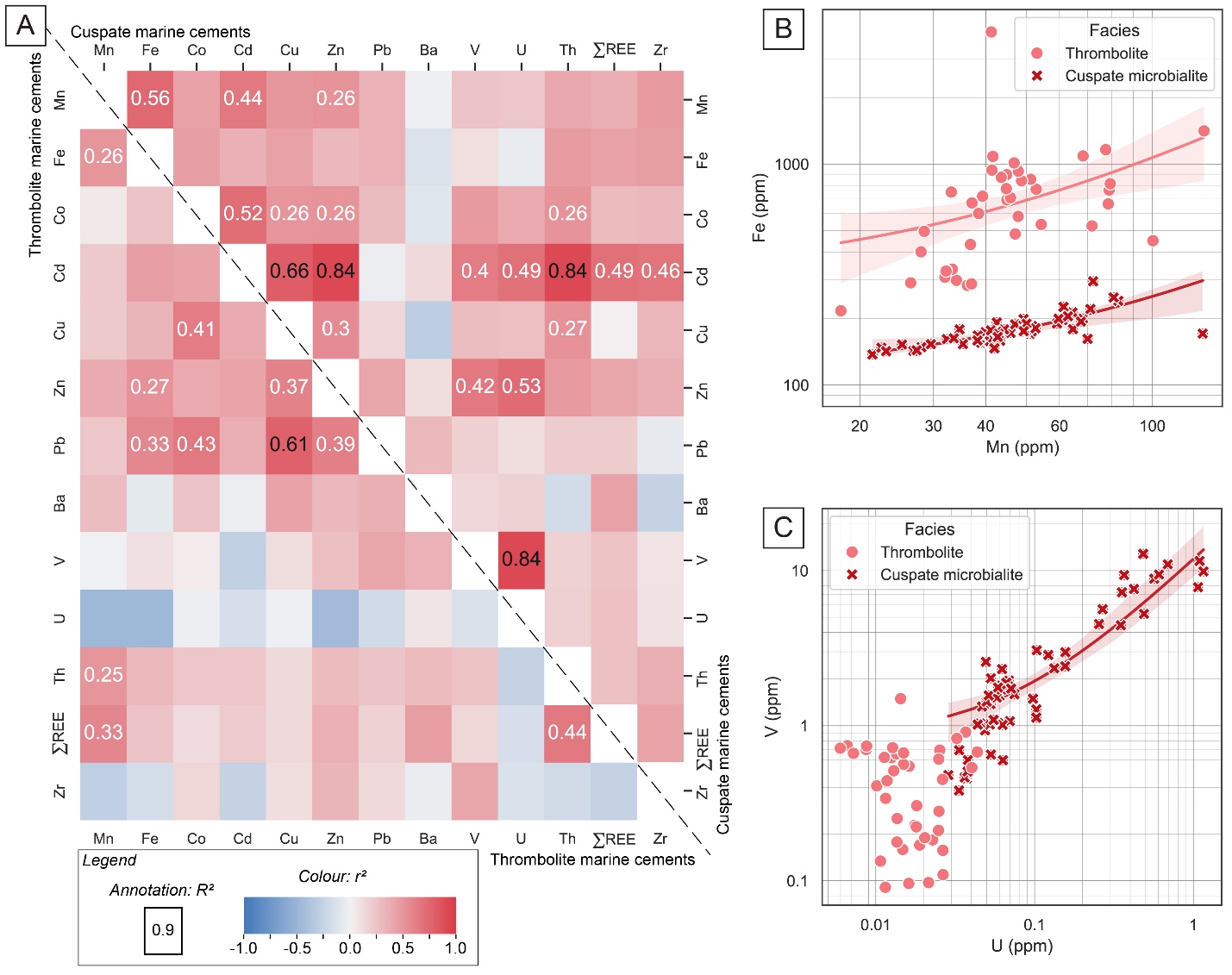


*Figure S6:* Correlation results. (A) Correlation matrix for the trace element concentrations of marine cements of the Callison Lake Formation. The lower half of the matrix is for the thrombolite facies, and the upper half of the matrix is for the cuspate microbialite facies. Cell colour is determined by the correlation coefficient, and cells are annotated with the coefficient of determination (R^2^) if R^2^ > 0.25. (B, C) Crossplots of Fe-Mn and V-U for marine cements in each facies of the Callison Lake Formation. Lines represent the linear regression model fit to the data; shading represents the 95% confidence interval of this regression fit.


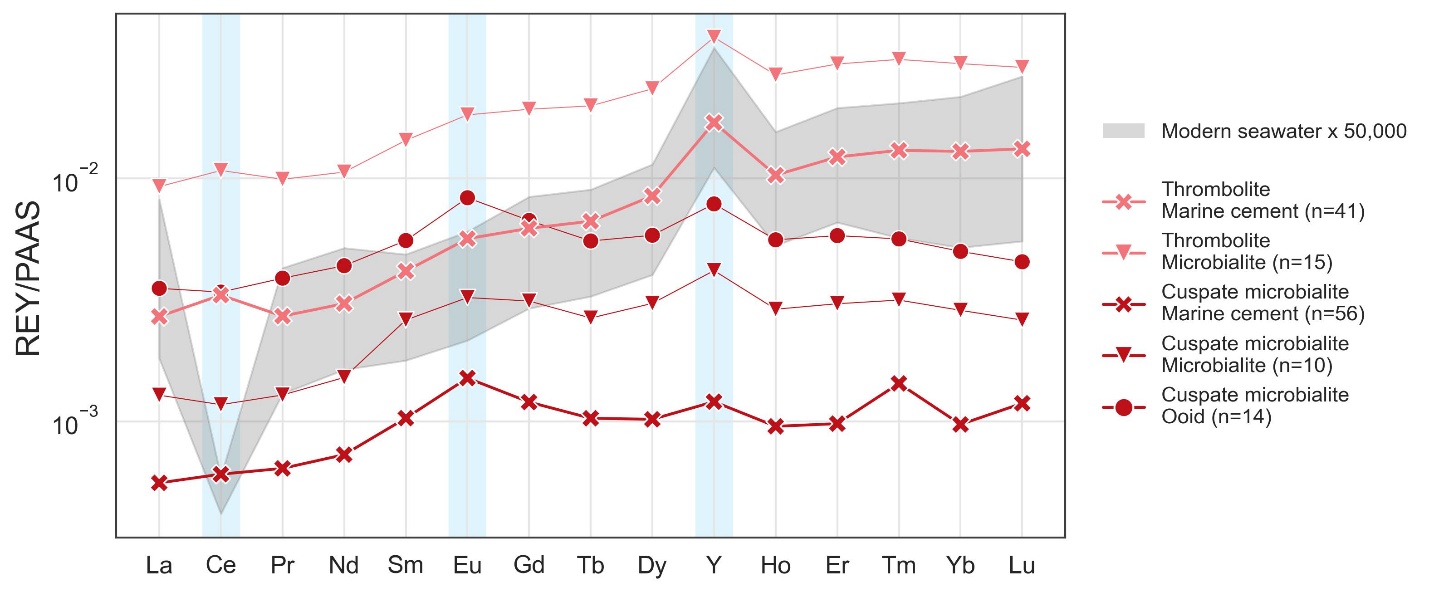


*Figure S7:* Average rare earth element plus Yttrium (REE+Y) distribution patterns for analyzed components in Callison Lake Formation microbialites, normalized to post-Archean average shale (McLennan, 1989). Results are from marine cements and microbialite in the thrombolite facies, and marine cements, microbialite and ooids in the cuspate microbialite facies. The grey background trace denotes 50,000x REE concentrations from modern Pacific seawater at a range of depths (Kamber, 2010).

| **Facies 1** | **Facies 2** | **Fe** | **Co** | **Zn** | **Cd** | **Pb** |
| --- | --- | --- | --- | --- | --- | --- |
| Callison Lake thrombolite | Callison Lake cuspate microbialite | 0.0000 | 0.0000 | 0.0000 | 0.0000 | 0.0199 |
| Callison Lake thrombolite | Nuccaleena shallow water | 0.6277 | 0.0000 | 0.0000 | 0.0000 | 0.0000 |
| Callison Lake thrombolite | Balcanoona deep water | 0.0000 | 0.0000 | 0.0000 | 0.2090 | 0.0000 |
| Callison Lake thrombolite | Beck Spring shallow water | 0.0000 | 0.0507 | 0.0000 | 0.8747 | 0.1130 |
| Callison Lake cuspate microbialite | Nuccaleena shallow water | 0.0000 | 0.0000 | 0.0000 | 0.0000 | 0.0000 |
| Callison Lake cuspate microbialite | Balcanoona deep water | 0.0000 | 0.0000 | 0.0000 | 0.0000 | 0.0000 |
| Callison Lake cuspate microbialite | Beck Spring shallow water | 0.7381 | 0.0016 | 0.3334 | 0.0009 | 0.8738 |

*Table S2:* Pairwise Welch’s unequal-variances *t*-tests comparing primary marine cement concentrations from different Neoproterozoic formations. Values shown are *p*-values for the null hypothesis of equal means.
